# Spectroscopic characterization, DFT, antimicrobial activity and molecular docking studies on 4,5-bis[(E)-2-phenylethenyl]-1H,1′H-2,2′-biimidazole

**DOI:** 10.1016/j.heliyon.2024.e29566

**Published:** 2024-04-16

**Authors:** M. Kiruthika, R. Raveena, R. Yogeswaran, N. Elangovan, Natarajan Arumugam, R. Padmanaban, Sinouvassane Djearamane, Ling Shing Wong, Saminathan Kayarohanam

**Affiliations:** a Department of Chemistry, Arignar Anna Government Arts College, Affiliated to Bharathidasan University, Musiri, 621211, Tiruchirappalli, Tamilnadu, India; b Research Centre for Computational and Theoretical Chemistry, Musiri, Anjalam, 621208, Tiruchirappalli, Tamilnadu, India; c Department of Chemistry, College of Science, King Saud University, P.O. Box 2455, Riyadh, 11451, Saudi Arabia; d Department of Chemistry, School of Physical, Chemical & Applied Sciences, Pondicherry University, R.V. Nagar, Kalapet, Puducherry, 605 014, India; e Department of Allied Health Sciences, Faculty of Science, Universiti Tunku Abdul Rahman, Jalan universiti, Bandar Barat, Kampar, 31900, Malaysia; f Biomedical Research Unit and Lab Animal Research Centre, Saveetha Dental College, Saveetha Institute of Medical and Technical Sciences, Saveetha University, Chennai, 602105, India; g Faculty of Health and Life Sciences, INTI International University, Nilai, 71800, Malaysia; h Faculty of Bioeconomics and Health Sciences, University Geomatika Malaysia, Kuala Lumpur, 54200, Malaysia

**Keywords:** Synthesis, DFT, NCI, Topology, Antimicrobial, Solvation, Human health

## Abstract

The newly synthesized imidazole derivative namely, 4,5-bis[(E)-2-phenylethenyl]-1H,1′H-2,2′-biimidazole (KA1), was studied for its molecular geometry, docking studies, spectral analysis and density functional theory (DFT) studies. Experimental vibrational frequencies were compared with scaled ones. The reactivity sites were determined using average localized ionization analysis (ALIE), electron localized function (ELF), localized orbital locator (LOL), reduced density gradient (RDG), Fukui functions and frontier molecular orbital (FMO). Due to the solvent effect, a lower gas phase energy gap was observed. Through utilization of the noncovalent interaction (NCI) method, the hydrogen bond interaction, steric effect and Vander Walls interaction were investigated. Molecular docking simulations were employed to determine the specific atom inside the molecules that exhibits a preference for binding with protein. The parameters for the molecular electrostatic potential (MESP) and global reactivity descriptors were also determined. The thermodynamic characteristics were determined through calculations employing the B3LYP/cc-pVDZ basis set. Antimicrobial activity was carried out using the five different microorganisms like *Escherichia coli*, *Streptococcus pneumoniae*, *Staphylococcus aureus*, *Klebsiella pneumoniae* and *Candida albicans*.

## Highlights

• Synthesis of new class of 4,5-bis[(E)-2-phenylethenyl]-1H,1′H-2,2′-biimidazole.
• Electronic properties were analysed by UV–Visible spectrum and FMO analysis.
• The active sites and interaction were determined using MEP, ELF, LOL, and RDG.
• The synthesized compound displayed moderate antimicrobial activity.
• Docking experiment showed the compound had significant high binding affinity score.

## 1 Introduction

Imidazole is a prominent heterocyclic molecule that is identified by its fundamental structure. This compound has garnered significant attention in the scientific community due to its wide range of applications, which can be attributed to the presence of nitro groups within its chemical structure. Imidazole exhibits a diverse range of biological actions, including antibacterial properties, antagonistic effects, anti-cancer activity, antiviral and anti-inflammatory properties, antioxidant effects, antifungal activity, and cytotoxicity [1]. This particular imidazole exhibits significant utility in several natural substances, including histamine, algacidal agents, and pilocarpine alkaloids. The imidazole framework encompasses several pharmaceutical compounds, including losartan (used for antihypertensive purposes), etomidate (a hypnotic medication), and flumazenil [2]. These pharmaceutical substances are widely distributed and utilized. Given the numerous applications of imidazole, researchers are currently dedicating their efforts towards the synthesis of further imidazole derivatives. Based on the aforementioned assessment, the crucial aspect lies in the advancement of a more pragmatic and adaptable approach utilizing the existing resources [3].

Khodja et al. just published a study detailing the design, synthesis, and biological evaluation of imidazole derivatives [4]. In a recent study, Kandasamy et al. (2020) presented their findings on the development of zinc binding groups derived from imidazole inhibitors, which have demonstrated potential in targeting lung cancer [5]. A recent publication has documented the utilization of imidazole as the foundation for the green production of an ionic liquid. The authors Shahi et al., provided an analysis of the synthesis and dispersion of electron density in imidazole derivatives [6].

In their study, Puratchikody and Doble provided a comprehensive account of the synthesis and pharmacological assessment of antinociceptive and anti-inflammatory properties in relation to 2-substituted-4,5-diphenyl-1H-imidazoles [7]. The evaluation of antinociceptive effects was conducted using hot plate and tail flick tests. Additionally, the authors conducted quantitative structure-activity relationship (QSAR) analyses to further investigate the relationship between chemical structure and biological activity [7]. Driven by the aforementioned findings, and as a component of our ongoing research initiative in the realm of computation, our objective is to produce a unique molecule known as KA1. In order to consider the reactivity and biological significance of the target compound, various analyses were conducted at the B3LYP/cc-pVDZ level of theory. The titled compound molecular docking was also done.

## 2 Experimental

### 2.1 Material and methods

The chemicals benzaldehyde, piperidine, and imidazole-2-carboxaldehyde were bought from Sigma-Aldrich. In contrast, solvents were readily available for purchase from local chemical suppliers to be utilized without any intermediate steps. The infrared spectra were analysed using KBr pellets and an Agilent spectrometer, with a measurement range of 4000-400 cm^−1^. The NMR spectrum was acquired using a Bruker-400 NMR instrument, with DMSO as the solvent. The UV–Visible spectrophotometer was used to record the absorption spectrum and Lambda-35 spectrophotometer were used to record the for-fluorescence spectrum.

### 2.2 Synthesis of (1E,5E)-1,6-diphenylhexa-1,5-diene-3,4-dione

Benzaldehyde (4.24 g, 40 mm) was added to a stirred solution of piperidine (2.0 mL, 20 mm) in 30 mL of methanol, and the resulting mixture was heated to reflux. Subsequently, a heated methanolic solution containing 2,3-butanedione (1.72 mL, 20 mm) was gradually introduced over a period of 2 h. The reflux process was extended for an additional duration of 3 h. The solution was allowed to cool to ambient temperature and thereafter stored in a refrigerator for the duration of the night. The dark violet precipitate was subjected to filtration, followed by washing with cold ethanol in three separate 15 mL portions. The resulting product, 4,5-bis[(E)-2-phenylethenyl]-1H,1′H-2,2′-biimidazole, was subsequently dried. The yield obtained from the experiment was determined to be 4.93 gm, which corresponds to a percentage yield of 47 %.

### 2.3 Syntheses of 4,5-bis[(E)-2-phenylethenyl]-1H,1′H-2,2′-biimidazole

**Fig. 1:**
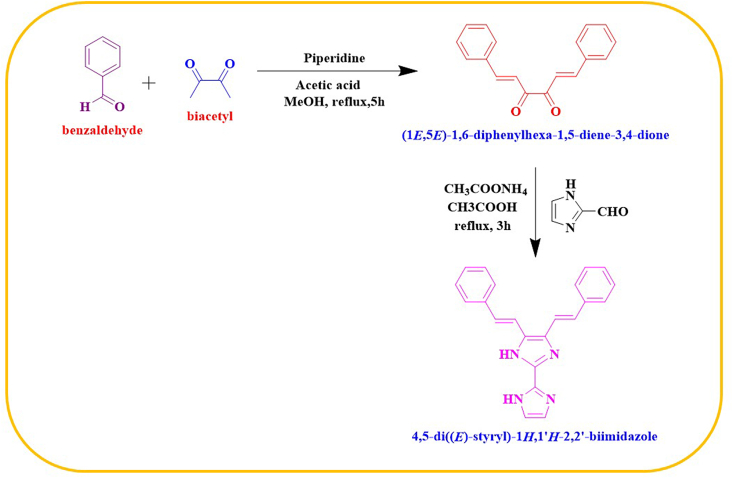
Syntheses of 4,5-bis[(E)-2-phenylethenyl]-1H,1’H-2,2′-biimidazole (KA1).

A mixture of (1E,5E)-1,6-diphenylhexa-1,5-dione (0.5 gm, 25 mm) and imidazole-2-carboxaldehyde (0.13 gm, 1.04 mm) was subjected to reflux for a duration of 3 h. Following the cooling process, a volume of 10 mL of cold water was introduced into the solution, resulting in the formation of an orange precipitate as the final product. The product was obtained as an orange precipitate through the process of column chromatography on silica, after undergoing filtration and purification [8]. Reaction scheme is presented in Fig. 1. The yield obtained from the experiment was determined to be 0.15 gm, corresponding to a percentage yield of 42 %.

### 2.4 Antimicrobial assay

The agar well diffusion method is a commonly employed technique for assessing the antimicrobial activity against various pathogens [9]. The evaluation of antimicrobial efficacy can be accomplished through the utilization of Muller Hinton Agar medium (HIMEDIAM173). Following the process of solidification, aseptic techniques were employed to create wells on each plate using a sterile corn borer with a diameter of 8 mm [10]. These wells were spaced 20 mm away from one another, with a total of four wells per plate. The test organisms standardised inoculum was evenly distributed on the solidified media surface using a sterile cotton swab. The necessary quantities of the sample were added to the first two wells, with test volumes of 10 μL, 30 μL, 60 μL respectively [11]. Gentamycin as a positive control, while the other well contained DMSO as a negative control. Subsequently, the agar plates were placed within an incubator set at a temperature of 37 °C for a duration of 24 h.

### 2.5 Molecular docking procedure

Lamarckian genetic algorithm (LGA) determined docking arrangement. Root-mean-square positional deviation and conformation similarity were used to group the conformations [12]. For the titled compound, run count was 10. The RMSD was calculated using a 2.0 Å conformational clustering tolerance [13]. A population size of 150, a mutation rate of 0.02, a crossover rate of 0.8, 27000 generations, and 2500000 energy evaluations were specified for the genetic algorithms. LGA docking used pseudo-Solis and Wets local search with 300 iterations each search [14]. The probability of a local search on a population member was 0.06. The maximum number of consecutive successes or failures before doubling or halving ρ was four for both. Finally, the lower constraint for ρ was fixed to 0.01.

### 2.6 Computational

The Gaussian software was utilized to optimize the KA1 structure [15]. During optimization B3LYP/cc-pVDZ basis set was utilized. Several calculations were done on the molecule optimized structure [16]. These calculations included infrared, Mulliken population analysis (MPA), natural population analysis (NPA), frontier molecular orbital analysis (FMOs), non-linear optical (NLO), molecular electrostatic potential (MESP) and natural bond orbital analysis (NBO) [17]. The HOMO-LUMO and MESP were conducted using the gas phase, water and chloroform. The potential energy distribution analysis (PED) calculations with the VEDA-4.0 software and visualization with the Gauss View 06 programme were used to give vibrational modes with a high level of accuracy [18]. Several studies, such as ELF, RDG, and LOL, were done with the help of the Multiwfn 3.4.1 [19]. The output data from Multiwfn were used with the VMD 1.9.1 programme to make the isosurface maps [20]. The auto-dock programme was used on the molecular docking study [21].

## 3 Results and discussion

### 3.1 Structural geometry analysis

**Fig. 2:**
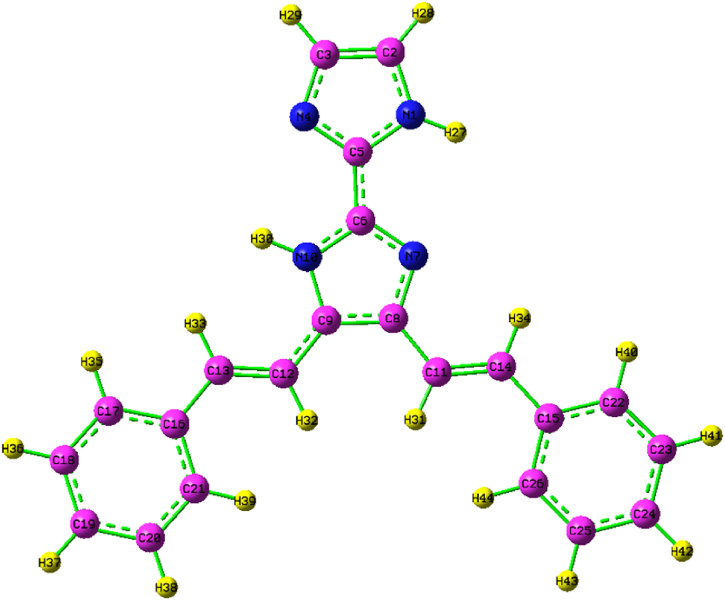
Optimized structure of 4,5-bis[(E)-2-phenylethenyl]-1H,1′H-2,2′-biimidazole.

The molecular geometry of the KA1 molecule was calculated using the Gaussian program. The optimized structural parameters obtained by the B3LYP/cc-pVDZ basis set method, and physical limitations are listed in Table S1. The KA1 molecule optimized structure is shown in Fig. 2. The lengths of the CC/CN bonds exhibit a high level of concordance with the existing data pertaining to molecules of comparable nature [18]. The observation reveals the presence of aberrations in the assessed geometrical parameters from the expected norm [22]. The observed changes can reasonably be attributed to the intermolecular interactions that exist in the crystalline state of the molecule [23]. The bond length of the C–C bond in the ring ranges from 1.35 to 1.46 Å, whereas the bond length of the C–H bond ranges from 1.09 to 1.09 Å. The highest bond length is C14–C15 (1.46 A°) and the lowest bond length is N1–H27 (1.01 A°). The same bond lengths are observed in N1–C2, C3–N4 and N7–C8, its bond length is 1.37 A°. The highest bond angle is C3–C2–H28 (132.43 A°) and the lowest bond angle is C8–C9–N10 (104.42 A°) [24,25]. The same bond angles are observed in C20–C19–H37, C25–C24–H24 (120.28 A°). The highest dihedral angle is C8–C9–C12–C13 (180.07 A°) and the lowest dihedral angle is N10–C9–C12–C13 (0.01 A°). The dihedral angles 1.79 A° is the same dihedral angles which is observed in C17–C16–C21–H39, C16–C17–C18–H36 and H37–C19–C20–C21 respectively.

### 3.2 FTIR analysis

**Fig. 3:**
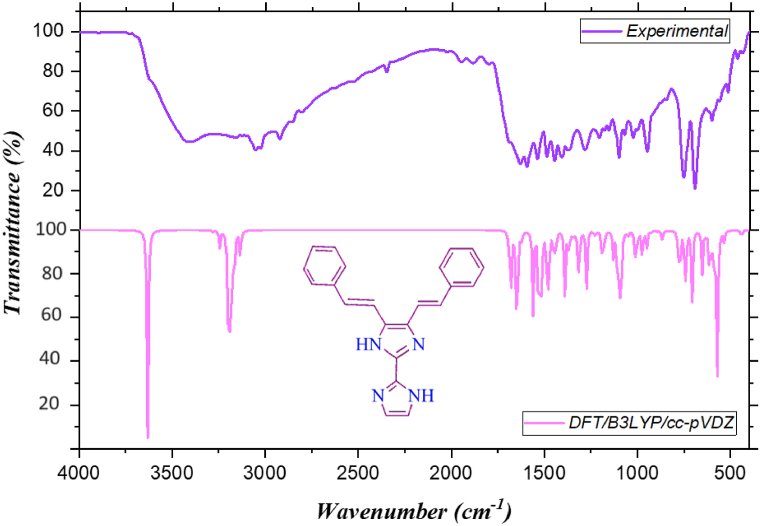
Comparison FTIR spectra of 4,5-bis[(E)-2-phenylethenyl]-1H,1′H-2,2′-biimidazole.

The FTIR analysis is conducted by concentrating on the distinctive vibrations shown by the C–C, C–H, and C–N groups. Table 1 provides a complete account of the observed IR and the theoretical IR vibrations [26]. The comparison FT-IR spectra of the titled compound are presented in Fig. 3. The theoretical wavenumbers were scaled in 0.9651 [27,28]. Theoretical and experimental wavenumbers exhibit a reasonable level of concurrence, and the allocation of wavenumbers to distinct functional groups is expounded upon in the subsequent discussion [29]. The compound KA1 having 44 atom and 126 modes of vibrations with C1 point group. The titled compound shows 43 stretching, 42 bending and 41 torsion vibrations.

**Table 1:** Comparison (experimental and theoretical) FTIR and PED analysis of 4,5-bis[(E)-2-phenylethenyl]-1H,1′H-2,2′-biimidazole.

B3LYP/cc-pVDZ					Observed	
Mode	Unscaled	Scaled	IR_I_	R_A_	IR	Assignment (% PED)
126	3632.54	3505.8	72.9089	44.9635	–	νNH(99)
125	3630.47	3503.8	75.4206	71.7009	3411	νNH(99)
124	3280.17	3165.7	0.9302	307.553	3161	νCH(75)
123	3244.93	3131.7	11.6048	187.265	–	νCH(75)
122	3200.9	3089.2	28.0372	478.054	–	νCH(82)
121	3199.25	3087.6	27.6882	393.828	–	νCH(81)
120	3192.43	3081	21.9702	144.338	–	νCH(86)
119	3190.49	3079.1	35.7845	70.8589	–	νCH(75)
118	3183.96	3072.8	5.2277	129.795	–	νCH(87)
117	3182.02	3071	6.5933	172.278	–	νCH(76)
116	3180.76	3069.8	24.7578	32.613	–	νCH(84)
115	3173.38	3062.6	3.9303	56.0651	–	νCH(82)
114	3171.72	3061	3.7541	122.553	–	νCH(83)
113	3168.97	3058.4	10.791	12.8492	–	νCH(80)
112	3165.81	3055.3	4.0776	44.1456	3056	νCH(81)
111	3164.55	3054.1	11.1755	43.5315	–	νCH(87)
110	3162.28	3051.9	2.2571	23.0565	–	νCH(88)
109	3133.64	3024.3	20.7163	25.5842	3028	νCH(98)
108	1688.87	1629.9	17.1644	5224.1	1633	νCC(51)
107	1677.71	1619.2	34.6924	13166.8	–	βHCC(10)+ νCC(42)
106	1656.46	1598.6	2.0582	4688.98	–	νCC(47)
105	1649.14	1591.6	41.9647	4057.7	1597	βHCC(22)+ νCC(49)
104	1645.55	1588.1	44.9783	13359.6	–	βHCC(14)+ νCC(31)
103	1622.05	1565.4	2.4973	886.927	–	βHCC((10)+ νCC(38)
102	1621.63	1565	4.5844	1539.1	1541	βHCC(12)+ νCC(21)
101	1561.02	1506.5	55.2879	1485.59	–	βHCC(38)
100	1533.71	1480.2	33.7216	550.279	1490	βHCC(61)
99	1524.26	1471.1	30.9353	145.801	–	βCNC(11)+ βHNC(13)+ νNC(10)
98	1515.48	1462.6	20.0848	1554.99	–	βHNC(18)+ νCC(26)
97	1513.88	1461	19.0749	1588.98	1449	βHCC(28)
96	1483.54	1431.8	61.9577	1024.84	–	βHCC(22)
95	1475.63	1424.1	3.5778	23.2914	–	νCC(44)
94	1470.1	1418.8	9.1397	159.947	1408	βHCC(25)+ νNC(11)
93	1449.25	1398.7	17.1677	546.697	–	βHCC(32)
92	1439.6	1389.4	15.9743	185.456	1372	βHCC(47)
91	1389.9	1341.4	42.6952	80.643	–	βHCN(13)
90	1369.39	1321.6	14.9574	28.054	–	βHCC(22)+ νCC(14)
89	1362.67	1315.1	2.2046	68.1733	–	βHCC(20)+ νCC(11)
88	1353.34	1306.1	1.3243	169.744	–	βHCC(38)+ νCC(13)
87	1345.33	1298.4	2.1629	2371.84	–	βCCC(12)+ βHCC(11)
86	1340.58	1293.8	0.7011	500.083	–	βHCC(19)+ νNC(11)+ νCC(14)
85	1329.75	1283.3	3.7982	462.073	1286	νNC(22)
84	1314.96	1269.1	39.5668	1697.39	–	νCC(15)
83	1314.31	1268.4	2.9621	68.9661	–	νCC(24)
82	1274.09	1229.6	41.1031	482.921	–	βHCN(13)+ νNC(11)
81	1260.51	1216.5	0.3607	3647.2	1209	βHCC(10)
80	1231.07	1188.1	3.3567	1021.54	–	βHCC(30)+ νCC(11)
79	1222.84	1180.2	1.3415	664.46	1181	βHCC(28)+ νNC(12)
78	1195.53	1153.8	0.6831	691.045	–	νCC(27)
77	1193.17	1151.5	1.1274	583.045	1157	βHCC(17)+ νNC(11)+ νCC(12)
76	1187.72	1146.3	28.0239	395.558	–	βHCC(62)
75	1176.05	1135	3.7381	146.898	–	βHCC(43)+ νNC(10)
74	1170.66	1129.8	0.6196	11.0392	–	βHNC(17)+ νNC(18)
73	1169.92	1129.1	0.2282	20.2897	1102	βHCC(13)+ νNC(16)+ νCC(24)
72	1128.29	1088.9	17.9306	147.596	–	βHCC(23)+ νCC(39)
71	1107.96	1069.3	28.8971	122.294	1073	βHCC(11)
70	1101.8	1063.3	5.6074	7.2748	–	βCCN(40)+ νNC(24)
69	1099.48	1061.1	15.4563	4.2241	–	νCC(53)
68	1088.97	1051	75.3984	31.5942	–	βCCN(18)+ βHNC(18)+ νNC(20)
67	1050.96	1014.3	2.0942	89.8653	1027	βCCC(56)+ νCC(17)
66	1050.48	1013.8	1.8424	21.7201	–	βHCC(11)
65	1017.37	981.86	2.4134	474.866	–	νCC(36)
64	1012.36	977.03	14.5378	4.2785	–	βCCC(16)
63	1010.33	975.07	0.2045	310.485	–	τCCCC(12)+ τHCCC(37)
62	1009.43	974.2	0.3386	442.296	–	τHCCC(15)
61	1007.46	972.3	0.7914	3.6454	–	τHCCC(80)
60	999.92	965.02	12.0733	0.032	951	τHCCC(74)+ βCNC(16)+ νNC(13)
59	983.19	948.88	0.0115	0.1357	–	τCCCC(13)+ τHCCC(74)
58	982.84	948.54	0.0248	0.1797	–	τHCCC(20)
57	973.45	939.48	21.4235	0.1396	–	τHCCC(89)
56	949.75	916.6	11.697	31.0802	–	βCNC(56)
55	933.41	900.83	0.2237	8.7107	–	τHCCC(76)
54	930.71	898.23	0.2144	9.0384	–	βCCN(54)+ νNC(15)
53	925.48	893.18	2.1404	26.1494	–	τCCCC(11)+ τHCCC(78)
52	897.48	866.16	0.6033	398.675	–	τHCCC(71)
51	891.3	860.19	0.6385	22.1546	–	τCNCC(12)+ τHCNC(75)
50	874.29	843.78	0.5242	15.37	–	βCCC(28)+ νNC(18)
49	869.73	839.38	1.5401	21.3144	–	τHCCC(64)
48	865.46	835.26	6.4796	1.1427	–	βCCN(22)+ βCCN(12)
47	851.88	822.15	0.0305	5.9291	–	τHCCC(95)
46	848.87	819.24	0.0875	7.2554	–	τHCCC(91)
45	832.83	803.76	0.6584	9.8141	–	τNCNC(71)+ τCNNC(10)
44	785.26	757.85	3.3501	1.0475	755	βCCC(19)+ νCC(11)
43	773.58	746.58	43.3332	1.0384	–	τCCCC(14)+ τHCCC(11)
42	754.55	728.22	0.7042	8.5441	–	τCCCC(10)+ τHCCC(10)+ τHCCN(35)
41	749.34	723.19	7.8518	13.0056	–	τCCCC(10)+ τHCCC(10)+ βCCN(45)
40	740.01	714.18	38.1897	0.1782	–	βCCN(26)
39	732.59	707.02	0.1518	0.2811	–	τHCCC11)
38	706.57	681.91	45.9676	0.8865	695	τCNNC(41)+ τHNCC(75)
37	704.71	680.12	1.5306	2.2455	–	τCCCC(21)+ τHCCC(58)
36	695.1	670.84	0.24	0.9674	–	τCCCC(22)+ τHCCC(57)
35	651.55	628.81	28.9062	0.2396	–	τHNCC(80)
34	631.7	609.65	1.943	5.8259	–	βCCC(69)
33	630.67	608.66	0.0624	23.2347	603	βCCC(85)
32	612.36	590.99	37.9598	31.2277	–	βCCC(11)
31	595.49	574.71	10.6104	20.2635	–	τCNCC(65)+ τHCNC(12)
30	584.69	564.28	35.7465	2.0924	–	βCNC(41)
29	570.01	550.12	49.4433	1.9576	–	βCCC(46)
28	570	550.11	49.5655	1.9565	–	βCCN(23)+ βCNC(17)+ βCCC(20)
27	533.95	515.32	7.5238	0.3338	516	τCCNC(59)
26	512.95	495.05	0.0224	1.0533	–	τCCCC(48)
25	469.27	452.89	0.2427	3.269	465	βCCC(60)
24	447.23	431.62	0.2348	0.5007	440	τCCNC(56)
23	440.22	424.86	5.547	23.7205	–	βCCN(41)+ βCNC(10)
22	414.58	400.11	0.0105	0.0332	–	τCCCC(76)+ τHCCC(11)
21	413.8	399.36	0.0039	0.0332	–	βCCC(11)
20	381.39	368.08	1.8734	8.3369	–	βCCC(14)+ βCNC(15)
19	353.9	341.55	1.872	6.0586	–	βCCN(45)
18	302.72	292.16	0.1818	2.9788	–	τCNNC(56)
17	289	278.91	1.0223	5.3271	–	τCCNC(25)
16	252.82	244	0.1316	1.5419	–	τNCCN(52)+ τCNNC(15)
15	226.28	218.38	0.82	14.5857	–	τCCCC(53)
14	191.24	184.57	0.0201	2.4509	–	βCNC(15)
13	184.35	177.92	8.8987	1.0242	–	βCNC(54)+ βCCC(18)
12	171.13	165.16	0.0607	10.4121	–	βCCC(66)
11	150.75	145.49	5.9416	0.1661	–	τNCCN(11)+ τCNNC(52)
10	117.14	113.05	1.1428	0.2384	–	τCNNC(41)
9	108.26	104.48	4.9534	3.5411	–	βCCN(83)
8	94.43	91.134	0.4627	7.0893	–	τCNNC(57)
7	75.77	73.126	4.3214	3.5268	–	τCCNC(15)+ τNCNC(60)+ τHNCC(84)
6	50.98	49.201	1.4125	0.4453	–	τCNNC(12)+ τCCNC(49)
5	44.45	42.899	1.0095	8.1638	–	βCCN(82)
4	34.3	33.103	0.6409	0.7934	–	τCNNC(51)
3	27.76	26.791	0.0106	11.931	–	βCCC(90)
2	20.23	19.524	0.0012	8.0866	–	τCCCC(67)
1	18.41	17.767	0.0007	6.358	–	τCCCC(30)

### 3.3 C–N vibration

Identifying the C–N vibration poses a significant challenge due to the potential for the mixing of many vibrations within this spectral area. Generally, the C–N stretching vibration of 1342–1266 cm^−1^ is found aromatic rings [30]. The chemical under investigation exhibits a C–N stretching vibration which is detected in the infrared (IR) spectrum at wavenumbers of 1286 cm^−1^ and the theoretical values observed in the region of 1417, 1418, 1293, 1 289, 1229, 1180, 1151, 1135, 1129, 1129, 1063 and 1050 cm^−1^ and the PED contribution is 10 %, 11 %, 11 %, 22 %, 11 %, 11 %,11 %, 1 %,1 8 %, 16 %, 24 %, 20 % respectively [31]. The titled compound bending vibration theoretically observed at 1471, 1462, 1341, 1229, 1129, 1129, 1050 cm^−1^ respectively. The bending vibration PED contribution is 13 %, 18 %,12 %,13 %, 17 %, 10 %, 18 % respectively. Simulated torsion vibrations are detected at 860, 728, 681, 574, 73 cm^−1^, with PED contribution of 75 %,35 %, 75 %, 12 %,84 % respectively.

### 3.4 C–C vibration

The C–C stretching vibrational range from 1600 to 1585 cm^−1^ [32]. In this compound the observed C–C stretching vibration is detected at 1633 cm^−1^. The molecule under investigation exhibits a C–C stretching vibration (simulated) detected at 1629, 1598, 1591, 1588, 1565, 1565, 1462, 1424, 1321, 1315, 1306, 1293, 1269, 1268, 1188, 1153, 1151, 1129, 1088, 1061, 1014, 981, 754 cm^−1^ with PED contribution of 51 %, 41 %, 49 %, 31 %, 38 %, 21 %, 26 %, 44 %, 14 %, 11 %, 14 %, 15 %, 24 %, 11 %, 27 %, 12 %, 24 %, 39 %, 55 %, 17 %, 36 %, 11 % respectively. Typically, the infrared spectrum exhibits the simulated bending vibrations of 1268, 1014, 977, 843, 757, 609, 608, 590, 550, 550, 452, 399, 368, 165, 26, cm^−1^ with PED contribution of 12 %, 56 %, 16 %, 28 %, 19 %, 69 %,85 %, 11 %, 46 %, 20 %, 60 %, 11 %, 14 %, 66 %, 90 %, respectively. Simulated torsion vibrations are detected at 975, 948, 893, 746, 728, 723, 680, 670, 495, 400, 218, 19, 17 cm^−1^ with PED contribution of 63 %, 59 %, 53 %, 43 %, 42 %, 41 %, 37 %, 36 %, 26 %, 22 %, 15 %, 2 %, 1 % respectively.

### 3.5 C–H vibration

Aromatic compounds display many different spectral bands within the range of 3000–2500 cm^−1^, which can be attributed to the stretching vibrations of aromatic carbon-hydrogen (C–H) bonds [33]. The C-H stretching vibrations of the titled compound are experimentally observed at 3161, 3056 and 3028 cm^−1^. The theoretically observed C–H stretching vibrations are 3165, 3131, 3089, 3087, 3081, 3079, 3072, 3070, 3069, 3062, 3061, 3058, 3055, 3054, 3051 and 3024 cm^−1^, with PED contribution of 75 %, 75 %, 82 %, 81 %, 86 %, 75 %, 77 %, 76 %, 84 %, 82 %, 83 %, 80 %, 81 %, 87 %, 88 % and 98 % respectively. The in-plane bending vibration are observed at 1286, 1209, 1181, 1157, 1102, 1073 and 1027 cm^−1^ respectively. The out-of-plane bending is detected in the range of 1000-750 cm^−1^. In experimental section the out-plane bending vibration is detected at 951 and 755 cm^−1^ respectively. The observed out-of-plane bending vibrational frequencies, both experimental and theoretical, are determined to be comfortably within their respective characteristic range.

### 3.6 Absorption spectroscopy

**Fig. 4:**
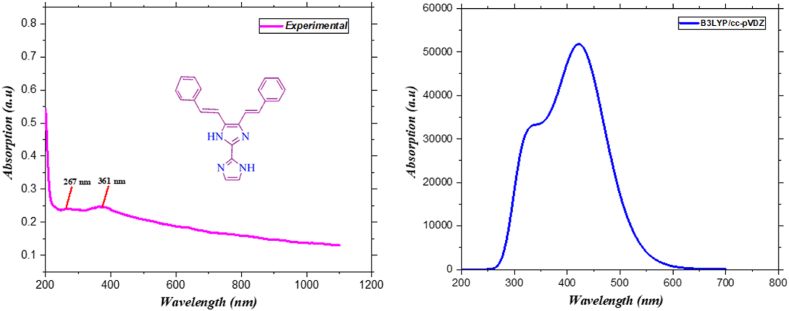
Comparison UV–Visible spectra of 4,5-bis[(E)-2-phenylethenyl]-1H,1′H-2,2′-biimidazole.

The absorption spectroscopic characteristics of the KA1 molecule were predicted using the time dependent density functional theory (TD-DFT) approach, the B3LYP/cc-pVDZ basis set, and the IEFPCM solvation model [34]. As the solvent in both the experiments and the theoretical analyses, acetonitrile was utilized. Table 2 presents the UV–Visible data derived through computational analysis [35]. The bonding, biological activity, and reactivity can be qualitatively studied by determining the HOMO and LUMO energy levels and the energy difference between them [36]. The energy gap of all FMO molecules is greater than 3 eV, as demonstrated by the data. The KA1 molecule possesses absorption maxima at 318, 364, and 430 nm, with oscillator strengths of 0.63, 0.51, and 1.16, respectively. The findings from the theoretical ultraviolet (UV) research provide confirmation that the predominant transitions observed are n–π* and π–π* transitions. The comparison experimental and simulated UV–visible spectrum are presented in Fig. 4. The molecule exhibits intramolecular charge transfer, which accounts for this phenomenon [37]. There were two different wavelengths of absorption observed in the experimental section, such as 267 nm and 361 nm, respectively, with oscillator strengths of 0.241 and 0.247.

**Table 2:** UV–Visible contribution, bond gap, energy and oscillator strengths of 4,5-bis [(E)-2-phenylethenyl]-1H,1′H-2,2′-biimidazole.

Media	Wavelength (nm)	Band Gap eV	Energy (cm-1)	Oscillator Strength	Contribution
**Chloroform**	430.1094035		23249.8986	1.1638	HOMO- > LUMO (100 %)
	364.6461476	3.1644	27423.8466	0.5142	HOMO- > L+1 (95 %), H-1- > LUMO (4 %)
	318.9035872		31357.4397	0.6319	H-1- > LUMO (90 %), HOMO- > L+1 (4 %), HOMO- > L+2 (2 %)

### 3.7 Emission spectroscopy

**Fig. 5:**
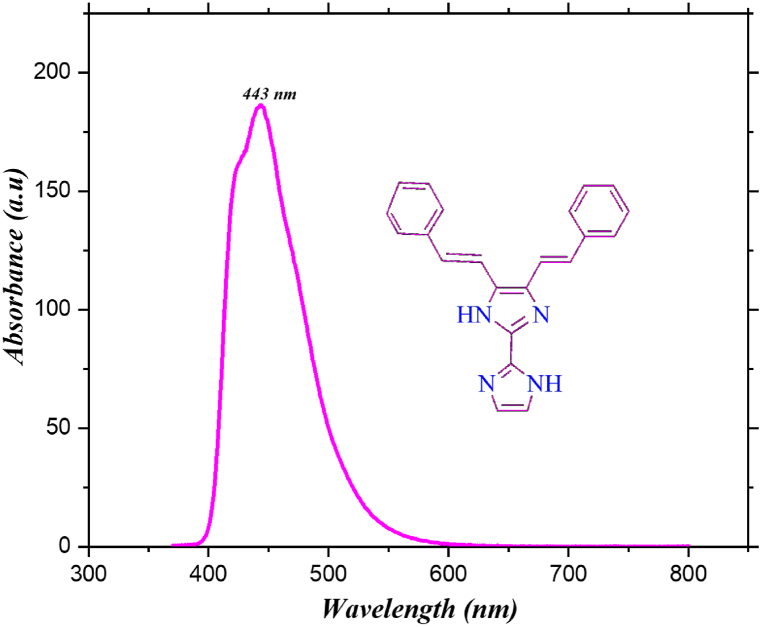
Fluorescence (emission) spectrum of 4,5-bis[(E)-2-phenylethenyl]-1H,1′H-2,2′-biimidazole.

The KA1 emission spectrum was captured in acetonitrile solvent, as shown in Fig. 5. A prominent band with an emission maximum about 443 nm is visible in the acetonitrile solvent spectra [38]. The lack of an emission band matching to the emission behaviour of follows Kasha's rule, which stipulates that only the excited state S1 is capable of emitting light among the excited states S1, S2, … Sn. As the polarity of the solvent increases, the emission maxima migrate towards the blue region [39]. In form, the fluorescence excitation spectrum closely resembles the absorption spectrum [40]. By determining the cross point (c) where the acetonitrile solvent absorption and emission spectra intersect, the S1 excitation energy (Es) may be computed.

### 3.8 NMR spectral analysis

**Fig. 6:**
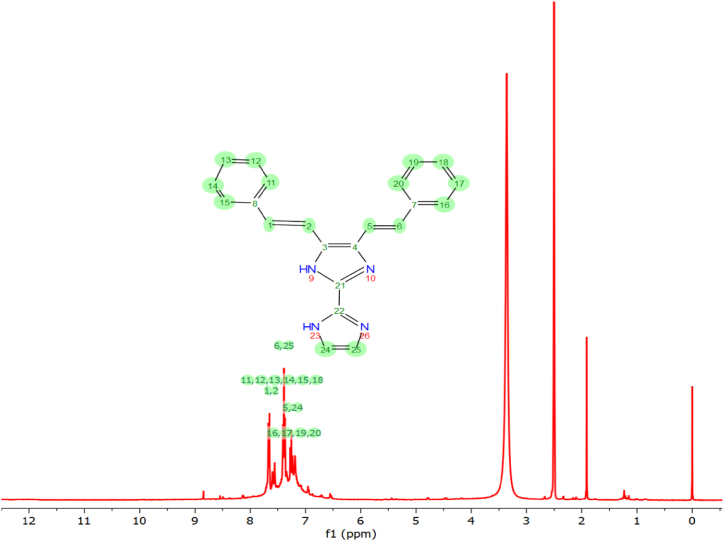
^1^HNMR spectrum of 4,5-bis[(E)-2-phenylethenyl]-1H,1′H-2,2′-biimidazole.

**Fig. 7:**
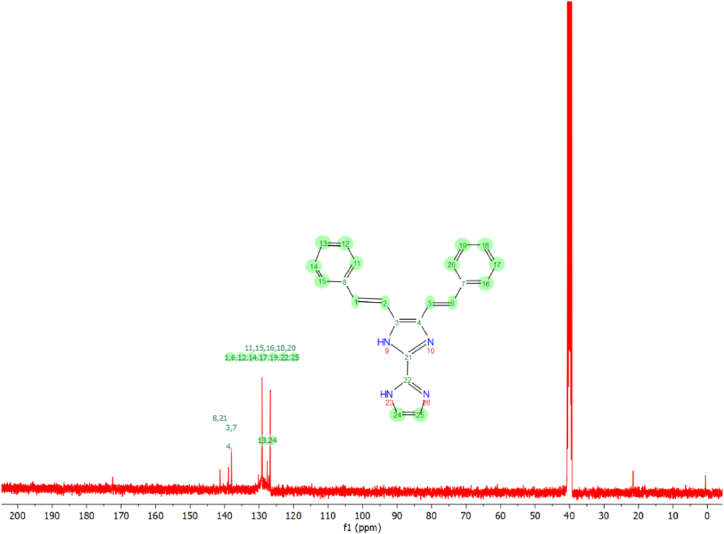
^13^CNMR spectrum of 4,5-bis[(E)-2-phenylethenyl]-1H,1′H-2,2′-biimidazole.

Fig. 6, Fig. 7 display the observed ^1^H and ^13^C NMR spectra of the compound KA1, recorded in a CDCl_3_ solvent. The theoretical chemical shift of KA1 was determined using the standard GIAO model [41]. To estimate the relative chemical shifts, the equivalent TMS shielding was used as a reference point. The TMS shielding was computed earlier at the same theoretical level. The chemical shift of protons typically seen within the range of 7.2–7.4 ppm, a characteristic that is commonly associated with protons located in phenyl rings and its theoretical calculated value is obtained for the range of 7.64–8.34 ppm [42]. The signals acquired at chemical shifts of 9.7, 7.6 and 7.4 ppm (1H-st,1H-dt and 1H-dt respectively) are indicative of the presence of five-member ring protons in the KA1 moieties and the theoretical values are noted at the range of 9.7, 7.6, and 7.5. The typical organic molecule exhibits a range of ^13^C NMR chemical shifts that typically exceeds 100 ppm. The high level of accuracy in these measurements permits the dependable interpretation of spectroscopic parameters [43]. The current investigation reveals that the ^13^C NMR chemical changes within the ring of KA1 exceed 100 ppm, aligning with the anticipated outcomes. The phenomenon of hybridization (namely sp3, sp2, sp) and the presence of electronegative groups (resulting from elements such as O, N, F, Cl, Br) give rise to significant shifts in the ^13^C chemical shifts. These shifts can be effectively employed to categorise various groups of resonances. In the context of resonance interactions within p systems, it is seen that smaller effects occurring within groups can lead to predictable up field and downfield chemical shift effects. In the current inquiry, the molecule being examined consists of three hydrogen atoms. The aromatic ring contains three atoms. The singlet proton labelled as H27 exhibits a significant downfield chemical shift in comparison to other protons [44]. This can be attributed to the presence of an electronegative nitrogen atom in close proximity. The measured chemical shift value exhibits a high level of concordance with the values obtained from calculations [45]. The aromatic carbon atoms inside the KA1 molecule were seen to exhibit chemical shifts ranging from 126.7 to 138 ppm, which aligns with the estimated values of 100.8–131.7 ppm. The carbon atom (C8 and C9) in the compound KA1, which formed bonds with nitrogen, has a measured ^13^C NMR shift of 138 and 129.1 ppm, falling within the anticipated range and the computational calculated values are 131.76 and 119.46 ppm. The chemical shift of carbon atom C6 is seen around 138.01 ppm and the DFT calculated value is 127.89 ppm which can be attributed to the high electronegativity of the next nitrogen atom. The chemical shift of the carbon atom at position C5 was determined to be 129.1 ppm.

### 3.9 FMO analysis

**Fig. 8:**
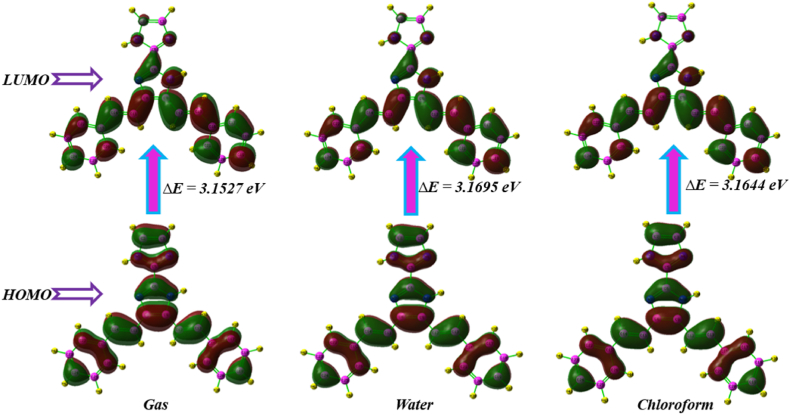
HOMO-LUMO energy diagram of 4,5-bis[(E)-2-phenylethenyl]-1H,1′H-2,2′-biimidazole in gas phase, water and chloroform.

The FMOs are the molecular orbitals associated with the FMOs energy level. These FMOs play a crucial role in elucidating the nature of a molecule's interactions with other species [46]. The HOMO exhibits electron-donating behaviour, while the LUMO demonstrates electron-accepting tendencies. The chemical stability and electron conductivity of a molecule can be evaluated by calculating the energy gap between their FMOs [47]. By connecting the electron donor groups at the molecule ends with the electron acceptor groups through a π-conjugated pathway, intramolecular charge transfer (ICT) occurs and a large energy gap is created [48]. DFT calculations were used to determine the HOMO and LUMO orbitals of the KA1 molecule. The cc-pVDZ basis set was employed for the calculations [49]. The resulting orbitals are depicted in Fig. 8. The observation indicates that the HOMO and LUMO exhibit delocalization across the whole of the molecule [50]. The research conducted by the FMOs reveals that the KA1 molecule exhibits charge delocalization, leading to an enhanced molecular reactivity. The HOMO and LUMO energies of KA1 were determined to be −4.99 eV and −1.84 eV in gas phase, furthermore water and chloroform HOMO and LUMO energies are −5.11 eV, −1.94 eV (water) and −5.06 eV, −1.90 eV (chloroform) respectively. The energy gap of HOMO and the LUMO was determined to be 3.15 eV (gas phase), 3.16 eV (water) and 3.16 eV (chloroform) respectively.

Table 3 presents the global reactive descriptor values of the titled compound. Molecular band gap energy directly affects chemical softness and hardness [51]. A molecule can be classified as hard if it possesses a significant energy gap, while a molecule with a tiny energy gap can be referred to as soft [52]. The polarizability of soft molecules is greater compared to that of hard molecules due to their lower energy need for excitation. The ionization potential (I) and electron affinity (A) values of the molecule KA1 are determined to be 4.99, 5.11, 5.06 eV and 1.84, 1.94, 1.90 eV corresponding to gas phase, water and chloroform respectively [53]. The determined values for the hardness (ƞ) and softness (S) of the KA1 molecule are 1.57, 1.58, 1.58 eV and 0.63, 0.63, 0.63 eV corresponding to gas phase, water and chloroform respectively [54]. The molecule under investigation has a notably low band gap energy, which is indicative of its stability.

**Table 3:** Frontier molecular orbital properties of 4,5-bis [(E)-2-phenylethenyl]-1H,1′H-2,2′-biimidazole in gas phase, water and chloroform.

Property	Gas	Water	Chloroform
ԑHOMO	−4.9965	−5.1132	−5.0667
ԑLUMO	−1.8438	−1.9437	−1.9023
Energy gap ΔE	3.1527	3.1695	3.1644
Ionisation energy (*I* = ԑHOMO = -HOMO)	4.9965	5.1132	5.0667
Electron Affinity (*A* = ԑLUMO = -LUMO)	1.8438	1.9437	1.9023
Global hardness (Ƞ = (*I-A*)/2)	1.5763	1.5847	1.5822
Global softness (*S = 1/Ƞ)*	0.6343	0.6310	0.6320
Chemical Potential (μ = -(I + *A*)/2)	−3.4201	−3.5284	−3.4845
Electronegativity (χ = -μ)	3.4201	3.5284	3.4845
Electrophilicity index (ω = μ2/2Ƞ)	3.0363	3.1408	3.0992
Nucleophilicity index (*N* = 1/ω)	0.3293	0.3183	0.3226
Electronaccepting powsr (ω+ = *A*2/2(*I-A*)	0.2924	0.3066	0.3005
Electrondonating power (ω+ = *I*2/2(*I-A*)	0.7924	0.8066	0.8005

### 3.10 MESP analysis

**Fig. 9:**
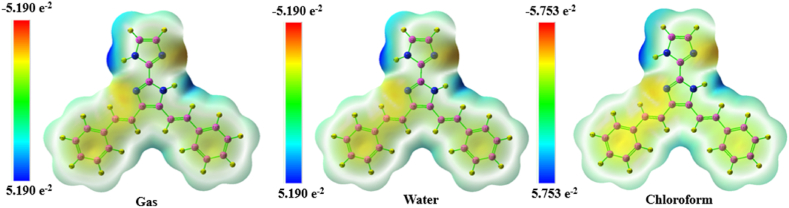
MESP surface map of 4,5-bis[(E)-2-phenylethenyl]-1H,1′H-2,2′-biimidazole in gas phase, water and chloroform.

The molecular electrostatic potential (MESP) characterises the overall electrostatic influence employed at a specific point in space by the combined charge distribution of a molecule, including both electrons and atomic cores [55]. By superimposing an electrostatic potential surface on top of an electron density isosurface, we may see information on the size, shape, charge density, and chemical reactivity sites of the molecule. Using Gaussian software and the cc-pVDZ basis set, we generate an electrostatic potential surface for the molecule [56]. Fig. 9 is presented a surface map of the MEP in gas phase and various solvent phase [57]. Electrostatic potential is shown in colours: red for negative, blue for positive, and green for neutral [14]. This creates areas with high negative electrostatic potential. The charge transfer within the cloud takes place between the range of −5.190 to +5.190 e^−2^ (gas phase), −5.753 to +5.753 e^−2^ (chloroform) and 5.190 to +5.190 e^−2^ (water) elementary charges. The calculated three-dimensional MEP reveals that the regions with negative electrostatic potential correspond to electrophilic areas, primarily attributed to the presence of nitrogen (N) atoms [58]. The nucleophilic sites in the molecule KA1 are primarily located on the hydrogen C–H atoms, which are considered as positive regions [59]. The use of the potential has mainly been observed in the prediction of sites and the assessment of relative reactivities towards electrophilic attack [60]. Additionally, it has been employed in investigations pertaining to biological recognition and interactions involving hydrogen bonding.

### 3.11 Natural population and Mulliken analysis

**Fig. 10:**
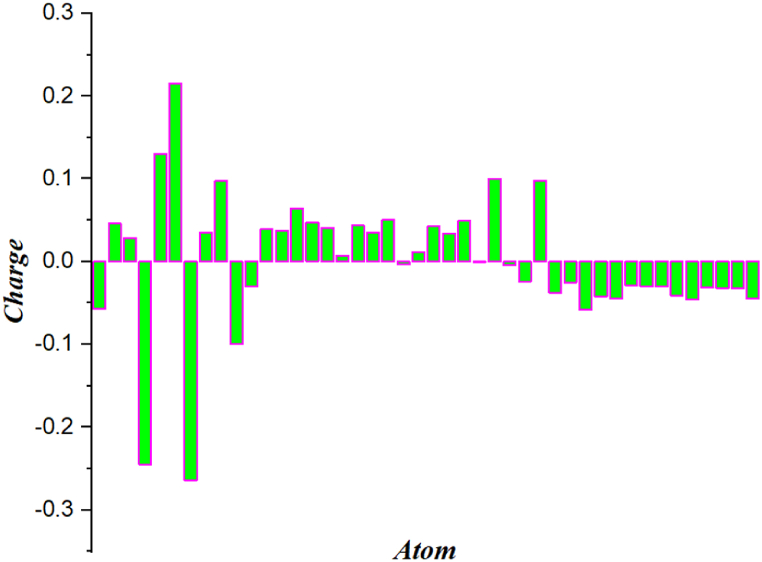
Mulliken analysis (MPA) of 4,5-bis[(E)-2-phenylethenyl]-1H,1′H-2,2′-biimidazole.

**Fig. 11:**
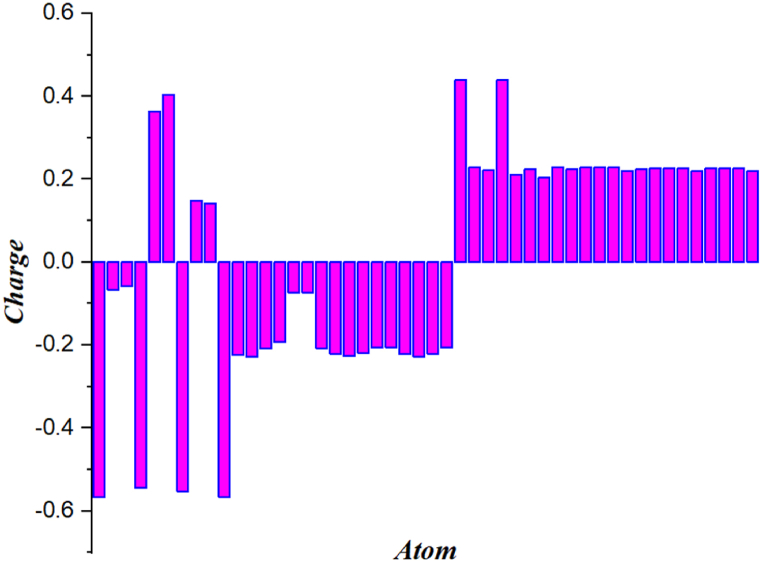
Natural population analysis (NPA) of 4,5-bis[(E)-2-phenylethenyl]-1H,1′H-2,2′-biimidazole.

Finding atomic charges in molecules such as MPA and NPA are the important one. Chemical electro negativity equalisation and charge transfer are characterised by atomic charge. Table 4, Table 5 show Mulliken and Natural atomic charges estimated using B3LYP/cc-pVDZ basis set [61]. The characteristic atomic charge affects quantum mechanical computations for molecular systems [62]. The MPA and NPA analyses are depicted in Fig. 10, Fig. 11 and values are presented in Table 4, Table 5 In the context of Mulliken, a significant majority of the charges exhibit a positive polarity. Certain atoms exhibit negative charges [63]. The atom with the most significant positive charge is C6 (0.21), while the atom with the most notable negative charge is C11 (−0.0304). All the nitrogen atoms contain negative charges only (N1/−0.05, N4/−0.24, N7/, −0.26, N10/−0.09). The N7 molecule has the highest negative charge, measuring −0.264 elementary charges, whereas the N1 molecule displays the smallest negative charge, measuring −0.05 elementary charges. The majority of carbon atoms possess a positive charge. Among these atoms, the carbon atom C12 exhibits the most pronounced negative charge, with a value of −0.03 [64]. Conversely, the carbon atom C26 displays the lowest negative charge, measuring −0.0007. All hydrogen atoms possess a both positive and negative charge, with each atom having a charge of around 0.10. In the context of natural charge, the C6 atom exhibited the most significant positive charge, measuring 0.43 elementary charges, whereas the N1 atom displayed the most substantial negative charge, measuring −0.56. The lowest positive charge was observed in C9 with a magnitude of 0.14 elementary charges, while the lowest negative charge was found in C3 with a magnitude of −0.05 elementary charges [65]. All hydrogen atoms possess a positive charge, with H30 (0.43) exhibiting a higher magnitude in comparison to other hydrogen atoms. There exist a pair of nitrogen atoms. Both possess a negative charge, with N1and N10 exhibiting a higher magnitude of negativity when compared to N4 and N7 respectively.

**Table 4:** Mulliken population analysis of 4,5-bis [(E)-2-phenylethenyl]-1H,1′H-2,2′-biimidazole.

Atom	Charge	Atom	Charge	Atom	Charge	Atom	Charge
1 N	−0.057095	12 C	0.039101	23 C	0.042563	34 H	−0.041894
2 C	0.046132	13 C	0.037584	24 C	0.033841	35 H	−0.044575
3 C	0.028413	14 C	0.06423	25 C	0.049073	36 H	−0.028466
4 N	−0.245092	15 C	0.046862	26 C	−0.000712	37 H	−0.030355
5 C	0.130661	16 C	0.040538	27 H	0.100349	38 H	−0.030066
6 C	0.215554	17 C	0.006978	28 H	−0.004212	39 H	−0.040999
7 N	−0.264238	18 C	0.043937	29 H	−0.024481	40 H	−0.045972
8 C	0.034581	19 C	0.034979	30 H	0.097798	41 H	−0.030781
9 C	0.097711	20 C	0.050582	31 H	−0.037231	42 H	−0.032594
10 N	−0.099911	21 C	−0.00322	32 H	−0.025243	43 H	−0.0327
11 C	−0.030418	22 C	0.011507	33 H	−0.058218	44 H	−0.044501

**Table 5:** Natural population analysis of 4,5-bis [(E)-2-phenylethenyl]-1H,1′H-2,2′-biimidazole.

	Natural	Natural Population			
Atom No	Charge	Core	Valance	Rydberg	Total
N 1	−0.56635	1.99921	5.55762	0.00952	7.56635
C 2	−0.06636	1.99904	4.04885	0.01847	6.06636
C 3	−0.0582	1.99907	4.03731	0.02182	6.0582
N 4	−0.5436	1.99942	5.52298	0.0212	7.5436
C 5	0.36431	1.99907	3.60413	0.03249	5.63569
C 6	0.40342	1.99906	3.56595	0.03158	5.59658
N 7	−0.55268	1.99938	5.53288	0.02042	7.55268
C 8	0.14926	1.99887	3.82689	0.02498	5.85074
C 9	0.14111	1.99884	3.8386	0.02145	5.85889
N 10	−0.56573	1.99915	5.55706	0.00952	7.56573
C 11	−0.22414	1.9989	4.2118	0.01344	6.22414
C 12	−0.22907	1.99891	4.21716	0.013	6.22907
C 13	−0.20786	1.99895	4.19284	0.01608	6.20786
C 14	−0.19226	1.99894	4.17625	0.01707	6.19226
C 15	−0.07247	1.99887	4.05828	0.01532	6.07247
C 16	−0.0728	1.99887	4.05837	0.01555	6.0728
C 17	−0.20698	1.99893	4.19363	0.01442	6.20698
C 18	−0.22054	1.99897	4.20689	0.01468	6.22054
C 19	−0.22606	1.99898	4.21233	0.01475	6.22606
C 20	−0.21818	1.99898	4.20451	0.01468	6.21818
C 21	−0.20641	1.99892	4.19375	0.01373	6.20641
C 22	−0.20478	1.99893	4.19141	0.01444	6.20478
C 23	−0.22243	1.99897	4.20882	0.01464	6.22243
C 24	−0.22727	1.99898	4.21355	0.01474	6.22727
C 25	−0.2205	1.99898	4.20684	0.01468	6.2205
C 26	−0.20543	1.99892	4.1928	0.01371	6.20543
H 27	0.43883	0	0.55562	0.00555	0.56117
H 28	0.22862	0	0.76926	0.00212	0.77138
H 29	0.22189	0	0.77623	0.00189	0.77811
H 30	0.43977	0	0.55486	0.00538	0.56023
H 31	0.21136	0	0.7852	0.00344	0.78864
H 32	0.22426	0	0.77244	0.0033	0.77574
H 33	0.20419	0	0.792	0.00381	0.79581
H 34	0.22936	0	0.76575	0.00489	0.77064
H 35	0.22349	0	0.77341	0.0031	0.77651
H 36	0.22863	0	0.76838	0.00298	0.77137
H 37	0.2278	0	0.7693	0.0029	0.7722
H 38	0.22805	0	0.76896	0.00299	0.77195
H 39	0.22091	0	0.77596	0.00314	0.77909
H 40	0.22452	0	0.77241	0.00307	0.77548
H 41	0.22717	0	0.76983	0.003	0.77283
H 42	0.2265	0	0.77058	0.00291	0.7735
H 43	0.22656	0	0.77043	0.00301	0.77344
H 44	0.22007	0	0.7768	0.00313	0.77993
H 41	0.22717	0	0.76983	0.003	0.77283
H 42	0.2265	0	0.77058	0.00291	0.7735
H 43	0.22656	0	0.77043	0.00301	0.77344
H 44	0.22007	0	0.7768	0.00313	0.77993

### 3.12 NBO analysis

NBO research helps examine a second-order interactions between occupied and valence sub cell orbitals. This study measures delocalization or hyperconjugation interactions [66]. Intramolecular hydrogen bonding, intramolecular charge transfer (ICT), and bond interactions have been studied using NBO analysis. The Gaussian 16 software package was used to compute the NBO [67]. The efficacy of the NBO analysis in facilitating the chemical interpretation of hyper conjugative interactions and the transfer of electron density from full lone pair electrons has already been demonstrated [68]. The interaction energy resulting from hyper-conjugation was determined using the second-order perturbation technique. The use of NBO theory extends to the identification of hydrogen bonding as well [69]. Table S2 presents the NBO analysis of the KA1. A significant contact has been noticed among the π-type orbital that encompasses the lone pair electron of N4 and N12, and the neighbouring σ*(C3–N12) and σ*(C6–C11) antibonding orbitals of the ring. These interactions result in a substantial stabilization energy of 10.36 and 11.69 kcal/mol respectively [70]. The electron density (ED) at the eight conjugated π bonds (with energies ranging from approximately 1.62 to 1.72 e) and π* bonds (with energies ranging from approximately 0.26 to 0.42 e) of the phenyl ring clearly demonstrates significant electron delocalization, leading to the stabilization of energy within the range of about 16.65–28.27 kJ/mol [13]. The phenomenon of electron delocalization is accountable for the molecular bioactivity and medicinal properties [71]. The titled compound highest stabilization energy was observed at bonding π(C18–C19, C16–C17, C20–C21, C25–C26) to antibonding π*(C20–C21, C18–C19, C16–C17, C23–C24) with stabilization energy of 20.28,21.31,19.54,19.55 kcal/mol respectively.

### 3.13 Topological analysis

The concept of electron localization function (ELF) refers to a theoretical approach used in quantum chemistry to describe the distribution and localization of electrons inside a molecular system [72]. The electron localization function (ELF) quantifies the probability of locating one electron in the immediate vicinity relative to another electron of identical spin. The aforementioned approach serves as a valuable means of determining the quantitative behaviour of electrons within a nuclear system [73]. It is widely utilized across a diverse range of applications, including the verification of geometric configurations and bond structures, elucidation of reactions leading to the formation of aromatic compounds, and other related purposes [74]. The diverse values of Extremely Low Frequency (ELF) are depicted through the utilization of distinct colour codes. The red colour is used to symbolise high values, while the blue colour is used to represent low values [75]. Supplementary Fig. 12 depicts the two-dimensional colour mapping of extremely low frequency (ELF) for the compound KA1. In the case of the compound under investigation, the highest level of Pauli repulsion was observed in the vicinity of the carbon (C) and nitrogen (N) atoms, as indicated by the red regions [76]. Conversely, the hydrogen atoms exhibited little Pauli repulsion, as evidenced by the blue regions around them.

**Fig. 12:**
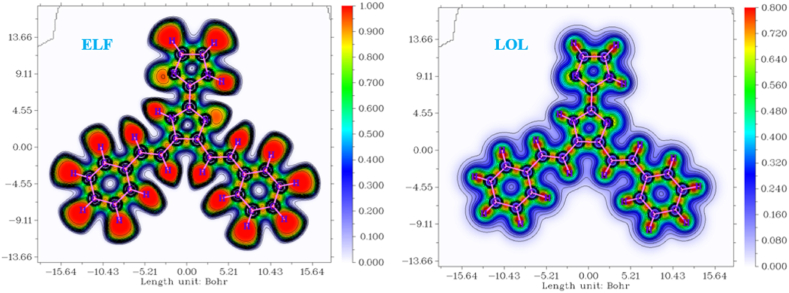
ELF and LOL surface map of 4,5-bis[(E)-2-phenylethenyl]-1H,1′H-2,2′-biimidazole.

The localised orbital locator is a technique utilized for the identification of electron localization. This localization is represented by a significant numerical value within the respective region. The approach bears resemblance to the ELF method. The observation of electron pair presence can be determined through the utilization of LOL. When localized orbitals come into contact, the gradient of their overlap is maximized [77]. The places on the LOL map with greater values indicate the dominance of electron localization over electron density, indicating the presence of lone pair electrons (covalent bond) or nuclear shell [78]. The map illustrating the location of the LOL compound is provided in Fig. 12. The graphic illustrates that the hydrogen atoms exhibit a significant electron density, while the carbon and nitrogen atoms display regions of comparatively lower electron density.

### 3.14 NCI analysis

**Fig. 13:**
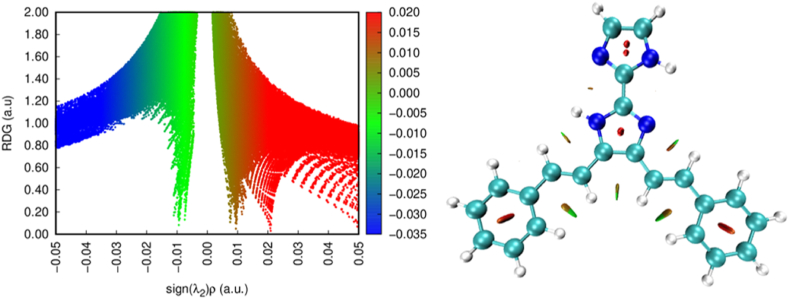
RDG surface map of 4,5-bis[(E)-2-phenylethenyl]-1H,1′H-2,2′-biimidazole.

The RDG is a fundamental dimensional parameter. The identification of the weak interaction is accomplished through the analysis of the electron density values, as depicted in Fig. 13. The plot depicting the relationship between RDG and r reveals the extent of interaction strength [79]. The k2 sign is utilized to distinguish between bonding interactions (k2 < 0) and non-bonding interactions (k2 > 0) [80]. The Multiwfn and VMD software are utilized for the analysis of the strength of interactions inside a molecular system [81]. In this analysis, the colour blue is indicative of stronger attraction, whereas the colour red represents repulsion [82]. This study reveals that the steric effect exhibits more prominence compared to other interactions, as seen by the red colour in the RDG scatter plot.

### 3.15 Antibacterial activity

**Fig. 14:**
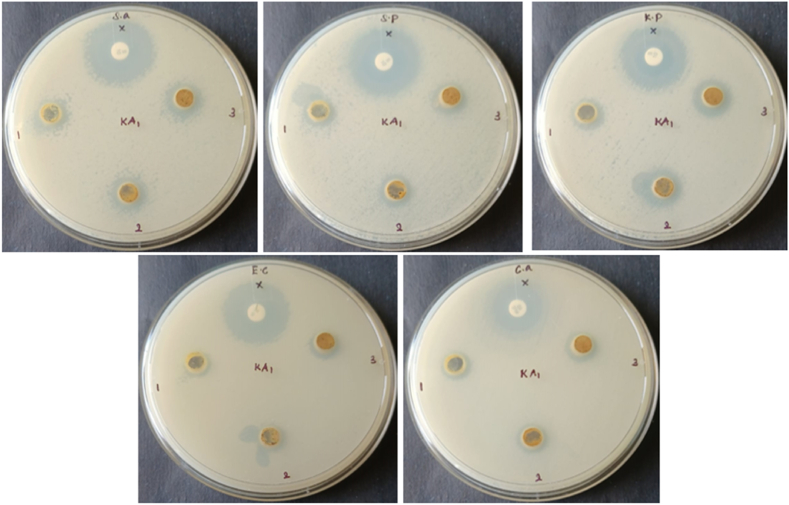
Antimicrobial activity - inhibition zone of 4,5-bis[(E)-2-phenylethenyl]-1H,1′H-2,2′-biimidazoleL(1), 30µL(2), and 60µL (3) and the positive control (x) towards *S.aureus, S. pneumoniaeS.aureus, S. pneumoniae, K. pneumoniae, E. coli and C. albicans respectively.*

The Muller Hinton Agar medium (HIMEDIA- M173) was employed for the assessment of microbiological susceptibility to antibacterial agents. The procedure involved the suspension of 38 g of a substance in 1000 mL of distilled water [83]. The temperature was maintained at a range of 45–50 °C. Thoroughly combine the components and carefully transfer the resulting mixture into aseptic petri dishes. The antibacterial efficacy of KA1was assessed against the four bacterial strains [84]. The obtained results were compared to the inhibition diameter of the positive control. The medication gentamicin exhibited variability in its extent of action. The KA1 compound exhibited a diameter of inhibition zone measuring 10.5, 12, 13.5, 14 mm against the *E.coli, S.pneumoniae, S.aureus, K.pneumoniae* respectively for 60 µL. This diameter of inhibition zone indicades that KA1 has the moderate antibacterial activity. The inhibition data for this drug are found in Table 6. The antibacterial activity is presented in Fig. 14.

**Table 6:** Antimicrobial activity of 4,5-bis [(E)-2-phenylethenyl]-1H,1′H-2,2′--biimidazole, diameter of inhibition zone in mm.

Organism	10 μl	30 μl	60 μl	Streptomycin
***S. aureus***	12	12.5	13.5	25
***S. pneumoniae***	11	11.5	12	26
***K. pneumoniae***	11.5	13.5	14	24
***E. coli***	9.5	10	10.5	23
				Clotrimazole
***C. albicans***	10	10.5	11	18

### 3.16 Antifungal activity

The study investigated the anti-fungal activity of KA1 against the fungal strain *C. albicans* [85]. The obtained results were compared to the inhibitory diameter of the positive control [86]. The findings indicate that the KA1 molecule exhibits notable anti-fungal property against *C. albicans*[87]. The inhibition data for this drug are found in Table 6. The antifungal activity is presented in Fig. 14.

### 3.17 Molecular docking study

**Fig. 15:**
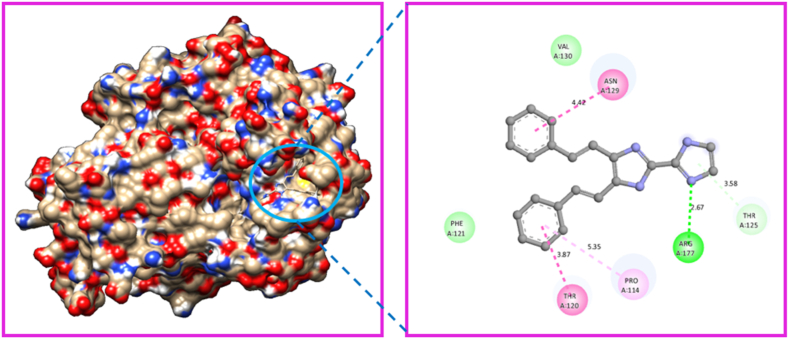
Protein-ligand interaction sites of 4,5-bis[(E)-2-phenylethenyl]-1H,1′H-2,2′-biimidazole with 6AKZ protein.

**Fig. 16:**
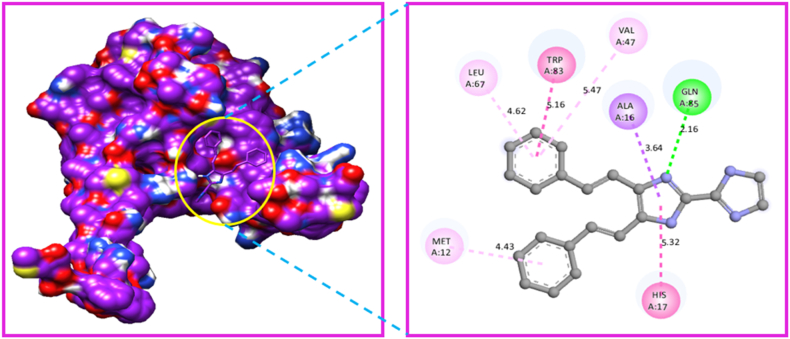
Protein-ligand interaction sites of 4,5-bis[(E)-2-phenylethenyl]-1H,1′H-2,2′-biimidazole with 4XB6 protein.

**Fig. 17:**
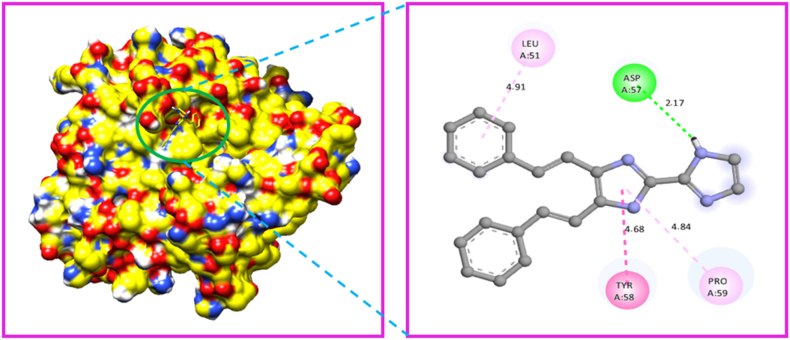
Protein-ligand interaction sites of 4,5-bis[(E)-2-phenylethenyl]-1H,1′H-2,2′-biimidazole with 4QLO protein.

**Fig. 18:**
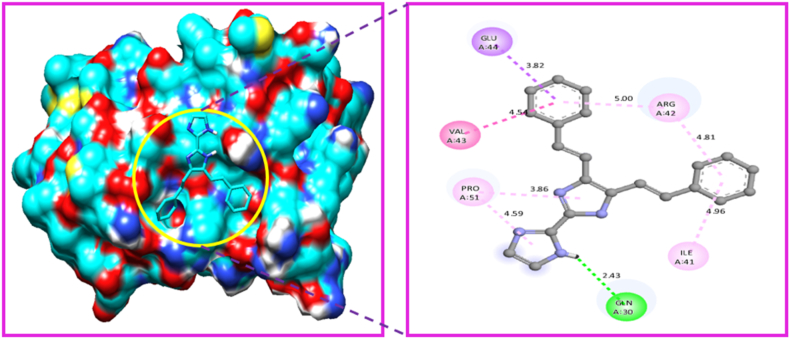
Protein-ligand interaction sites of 4,5-bis[(E)-2-phenylethenyl]-1H,1′H-2,2′-biimidazole with 4F2E protein.

**Fig. 19:**
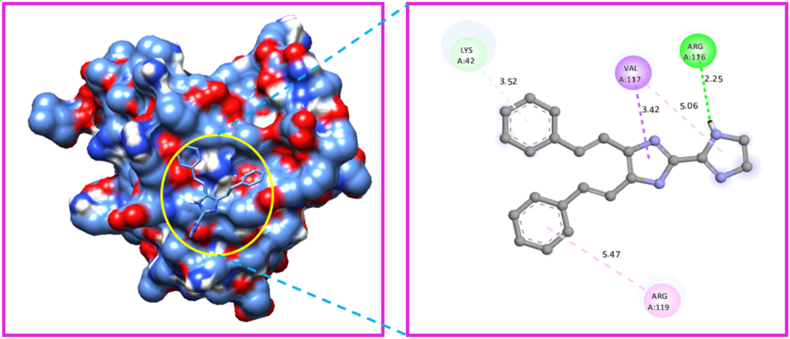
Protein-ligand interaction sites of 4,5-bis[(E)-2-phenylethenyl]-1H,1′H-2,2′-biimidazole with 4HWM protein.

The Gaussian programme was used to optimize the molecular structure of the molecule KA1. The molecular docking technique was carried out with the help of Auto-Dock Tools-1.5.4, which was included in the MGL Tools-1.5.4 package [88]. The current docking analysis was performed using different proteins includes 6AKZ 4XB6, 4QLO, 4F2E, and 4HWM. The protein was chosen based on the antimicrobial activity study. In antimicrobial activity analysis study, we took five different microorganisms, so we can choose five different proteins related to antimicrobial activity study. The proteins were downloaded from RCB (protein data bank) [89]. Several steps were taken to prepare protein. The crystal structure all water molecules were removed, than added polar hydrogen atoms, and coulomb charges. The Auto-dock software generated affinity grids with the highest grid size and centred on the active site [90]. The proteins that were chosen for analysis were subjected to molecular docking with the ligand. Among the selected target protein 6AKZ exhibited the most favourable binding energy, with a value of −6.95 kcal/mol. The ligand forms hydrogen bonds with the amino acid ARG-177 in the active site of the target protein through N–H ⋯ N interactions (Fig. 15). The protein 4XB6 exhibited a high affinity for the ligand, as evidenced by its binding energy of −6.40 kcal/mol [91]. Additionally, a hydrogen bond interaction was observed between the residue GLN-85 to the ligand (Fig. 16). The ligand KA1 was subjected to a docking process with the protein 4QLO. During this process, a single hydrogen bond (strong non-covalent type of interaction) was seen between the ligand and the amino acid ASP-57 [92]. The resulting binding energy was calculated to be −5.84 kcal/mol (Fig. 17). Now, the KA1 ligand was subjected to a docking process with the protein 4F2E. During this process, a single hydrogen bond was seen between the ligand and the amino acid GLN-30 [93]. The resulting binding energy was calculated to be −6.23 kcal/mol (Fig. 18). The ligand KA1 was subjected to a docking process with the protein 4HWM, during this process, a single hydrogen bond was seen between the ligand and the amino acid ARG-116 and the resulting binding energy was calculated to be −6.00 kcal/mol (Fig. 19). Table 7 presents the highest binding energies observed between chosen target proteins and their respective ligands [94]. Table 8 presents the non-bonded interaction of the ligand and targeted proteins. The low energy binding values are indicative of the ligand protein complex maximum affinity. The present study investigates the activity of the compound carbonitrile against protein 6AKZ. The results demonstrate that this compound exhibits the highest binding energy of −6.95 kcal/mol.

**Table 7:** Binding energies of 4,5-bis [(E)-2-phenylethenyl]-1H,1′H-2,2′-biimidazole with 6AKZ, 4XB6, 4HWM, 4F2E and 4QLO.

Proteins	Rank	Run	Binding Energy	Cluster RMSD	Reference RMSD
4QLO	1	9	−5.84	0	24.91
	1	8	−5.61	0.94	25.36
	1	7	−5.55	1.38	25.04
	1	1	−5.23	1.2	24.86
	1	3	−5.01	1.31	25.08
	2	10	−5.73	0	21.79
	3	6	−4.88	0	21.08
	4	4	−4.8	0	46.79
	5	5	−4.8	0	23.22
	6	2	−4.33	0	25.75
6AKZ	1	1	−6.95	0	77.85
	2	7	−6.09	0	87.25
	3	2	−6.03	0	81.17
	4	10	−5.81	0	40.4
	5	8	−5.52	0	44.09
	6	3	−5.29	0	46.86
	7	4	−5.08	0	84.51
	8	5	−5.04	0	41.88
	8	6	−4.86	1.35	41.65
	9	9	−4.92	0	63.25
4XB6	1	10	−5.61	0	43.54
	2	2	−4.94	0	39.91
	2	6	−4.34	1.8	41.06
	3	8	−4.9	0	34.8
	4	9	−4.42	0	42.83
	5	3	−4.2	0	38.84
	6	5	−4.14	0	44.26
	7	4	−4.14	0	47.28
	8	1	−4.09	0	57.19
	9	7	−3.86	0	35.15
4HWM	1	4	−6	0	39.55
	1	10	−5.97	1.98	39.9
	1	3	−5.95	1.67	38.95
	2	6	−5.75	0	39.51
	3	5	−5.58	0	41.65
	4	2	−5.46	0	34.79
	4	9	−5.34	1.17	33.85
	4	1	−5.27	0.77	34.21
	4	7	−5.21	1.22	34.4
	5	8	−5.03	0	23.31
4F2E	1	1	−6.23	0	17.92
	1	6	−6.17	1.18	18.08
	1	3	−6.16	0.19	17.94
	1	7	−6	1.18	17.92
	1	10	−5.91	1.53	17.7
	2	5	−5.83	0	27.76
	3	8	−5.66	0	25.63
	4	9	−5.51	0	30.28
	4	2	−5.45	1.85	29.98
	5	4	−5.31	0	24.64

**Table 8:** Protein-ligand non-bonded interactions of 4,5-bis [(E)-2-phenylethenyl]-1H,1′H-2,2′-biimidazole with 6AKZ, 4XB6, 4HWM, 4F2E and 4QLO.

Protein	Distance	Category	Type	From	From-Chem	To	To-Chem
6AKZ	2.67292	Hydrogen Bond	Conventional Hydrogen Bond	A:ARG177:HH1	H-Donor	:UNK0:N	H-Acceptor
	3.57562	Hydrogen Bond	Pi-Donor Hydrogen Bond	A:THR125:OG1	H-Donor	:UNK0	Pi-Orbitals
	3.86632	Hydrophobic	Amide-Pi Stacked	A:THR120:C,O; PHE121:N	Amide	:UNK0	Pi-Orbitals
	4.4191	Hydrophobic	Amide-Pi Stacked	A:ASN129:C,O; VAL130:N	Amide	:UNK0	Pi-Orbitals
	5.35168	Hydrophobic	Pi-Alkyl	:UNK0	Pi-Orbitals	A:PRO114	Alkyl
	2.15747	Hydrogen Bond	Conventional Hydrogen Bond	A:GLN85:HN	H-Donor	:UNK0:N	H-Acceptor
	3.64127	Hydrophobic	Pi-Sigma	A:ALA16:CB	C–H	:UNK0	Pi-Orbitals
	5.16026	Hydrophobic	Pi-Pi Stacked	A:TRP83	Pi-Orbitals	:UNK0	Pi-Orbitals
4XB6	5.31545	Hydrophobic	Pi-Pi T-shaped	A:HIS17	Pi-Orbitals	:UNK0	Pi-Orbitals
	5.46916	Hydrophobic	Pi-Alkyl	:UNK0	Pi-Orbitals	A:VAL47	Alkyl
	4.61615	Hydrophobic	Pi-Alkyl	:UNK0	Pi-Orbitals	A:LEU67	Alkyl
	4.42711	Hydrophobic	Pi-Alkyl	:UNK0	Pi-Orbitals	A:MET12	Alkyl
	2.25319	Hydrogen Bond	Conventional Hydrogen Bond	:UNK0:H	H-Donor	A:ARG116:O	H-Acceptor
	3.51556	Hydrogen Bond	Pi-Donor Hydrogen Bond	A:LYS42:O	H-Donor	:UNK0	Pi-Orbitals
4HWM	3.41535	Hydrophobic	Pi-Sigma	A:VAL117:CG1	C–H	:UNK0	Pi-Orbitals
	5.47372	Hydrophobic	Pi-Alkyl	:UNK0	Pi-Orbitals	A:ARG119	Alkyl
	5.05815	Hydrophobic	Pi-Alkyl	:UNK0	Pi-Orbitals	A:VAL117	Alkyl
	2.43278	Hydrogen Bond	Conventional Hydrogen Bond	:UNK0:H	H-Donor	A:GLN30:OE1	H-Acceptor
	3.82085	Hydrophobic	Pi-Sigma	A:GLU44:CB	C–H	:UNK0	Pi-Orbitals
	4.53503	Hydrophobic	Amide-Pi Stacked	A:VAL43:C,O; GLU44:N	Amide	:UNK0	Pi-Orbitals
4F2E	3.85896	Hydrophobic	Pi-Alkyl	:UNK0	Pi-Orbitals	A:PRO51	Alkyl
	5.00427	Hydrophobic	Pi-Alkyl	:UNK0	Pi-Orbitals	A:ARG42	Alkyl
	4.961	Hydrophobic	Pi-Alkyl	:UNK0	Pi-Orbitals	A:ILE41	Alkyl
	4.80818	Hydrophobic	Pi-Alkyl	:UNK0	Pi-Orbitals	A:ARG42	Alkyl
	4.59193	Hydrophobic	Pi-Alkyl	:UNK0	Pi-Orbitals	A:PRO51	Alkyl
	2.16594	Hydrogen Bond	Conventional Hydrogen Bond	:UNK0:H	H-Donor	A:ASP57:O	H-Acceptor
	4.67892	Hydrophobic	Amide-Pi Stacked	A:TYR58:C,O; PRO59:N	Amide	:UNK0	Pi-Orbitals
4QLO	4.84421	Hydrophobic	Pi-Alkyl	:UNK0	Pi-Orbitals	A:PRO59	Alkyl
	4.91312	Hydrophobic	Pi-Alkyl	:UNK0	Pi-Orbitals	A:LEU51	Alkyl

## 4 Conclusion

The experiments involving infrared, absorption, emission, ^1^H, and ^13^C NMR data demonstrate a strong agreement with the DFT calculations. The localization and delocalization of electrons were determined through the application of topological methods. Multiwfn is used to identify intramolecular hydrogen bonds. A minimum energy gap value was observed during the investigation of FMO that indicates a high stability of the specified molecule. According to the FMO analysis, the gas phase has the lowest bond gap value. Based on TD-DFT calculations, UV–Visible values for the relevant molecules are in significant agreement with the observed UV–Visible spectrum values. In its fluorescence spectrum, the molecule displayed a dominant wavelength at 443 nm. The MEP research indicate that both electrophilic and nucleophilic attacks are observed on the synthesized molecule. Molecular docking analysis revealed that the most significant protein-ligand interaction energy exists in the compound, as well as a favourable dipole moment and polarizability. The results might be useful for future development of drug to promote human health.

## Data Availability

Date will be made available on request.

## CRediT authorship contribution statement

**M. Kiruthika:** Validation, Methodology, Investigation, Data curation, Conceptualization. **R. Raveena:** Validation, Methodology, Formal analysis, Data curation. **R. Yogeswaran:** Visualization, Validation, Resources. **N. Elangovan:** Writing – review & editing, Writing – original draft, Validation, Methodology, Formal analysis, Data curation, Conceptualization. **Natarajan Arumugam:** Writing – review & editing, Writing – original draft, Project administration, Methodology, Investigation, Funding acquisition, Conceptualization. **R. Padmanaban:** Writing – review & editing, Writing – original draft, Validation, Software, Formal analysis. **Sinouvassane Djearamane:** Validation, Supervision, Software, Resources, Formal analysis, Data curation. **Ling Shing Wong:** Visualization, Validation, Project administration, Methodology, Investigation. **Saminathan Kayarohanam:** Visualization, Resources, Project administration, Investigation, Funding acquisition.

## Declaration of competing interest

The authors declare the following financial interests/personal relationships which may be considered as potential competing interests: Natarajan Arumugam reports was provided by King Saud University. If there are other authors, they declare that they have no known competing financial interests or personal relationships that could have appeared to influence the work reported in this paper.
