# Supplementary material for: Spectroscopic characterization, DFT, antimicrobial activity and molecular docking studies on 4,5-bis[(E)-2-phenylethenyl]-1H,1′H-2,2′-biimidazole
Source: Heliyon. 2024 Apr 16;10(9):e29566. doi: 10.1016/j.heliyon.2024.e29566 (PMC11066587; doi:10.1016/j.heliyon.2024.e29566)
Supplement: Multimedia component 1 [file mmc1.docx]

**Supplementary information**

**Table S1 Structural parameters of 4,5-bis[(E)-2-phenylethenyl]-1H,1’H-2,2’-biimidazole**

| **Bond** | **Bond Length, Angle, Dihedral Angle** |
| --- | --- |
| (N1,C2) | 1.3757 |
| (N1,C5) | 1.3655 |
| (N1,H27) | 1.0132 |
| (C2,C3) | 1.3812 |
| (C2,H28) | 1.0855 |
| (C3,N4) | 1.3738 |
| (C3,H29) | 1.0877 |
| (N4,C5) | 1.3275 |
| (C5,C6) | 1.4451 |
| (C6,N7) | 1.3233 |
| (C6,N10) | 1.3638 |
| (N7,C8) | 1.3799 |
| (C8,C9) | 1.4101 |
| (C8,C11) | 1.4483 |
| (C9,N10) | 1.3831 |
| (C9,C12) | 1.4377 |
| (N10,H30) | 1.0127 |
| (C11,C14) | 1.3534 |
| (C11,H31) | 1.0931 |
| (C12,C13) | 1.3571 |
| (C12,H32) | 1.0922 |
| (C13,C16) | 1.4635 |
| (C13,H33) | 1.096 |
| (C14,C15) | 1.4642 |
| (C14,H34) | 1.0942 |
| (C15,C22) | 1.4104 |
| (C15,C26) | 1.412 |
| (C16,C17) | 1.4107 |
| (C16,C21) | 1.4122 |
| (C17,C18) | 1.3952 |
| (C17,H35) | 1.0934 |
| (C18,C19) | 1.3974 |
| (C18,H36) | 1.0925 |
| (C19,C20) | 1.4008 |
| (C19,H37) | 1.0921 |
| (C20,C21) | 1.3927 |
| (C20,H38) | 1.0926 |
| (C21,H39) | 1.0917 |
| (C22,C23) | 1.3955 |
| (C22,H40) | 1.0932 |
| (C23,C24) | 1.3974 |
| (C23,H41) | 1.0926 |
| (C24,C25) | 1.4008 |
| (C24,H42) | 1.0922 |
| (C25,C26) | 1.3929 |
| (C25,H43) | 1.0927 |
| (C26,H44) | 1.0918 |
| (C2,N1,C5) | 107.2299 |
| (C2,N1,H27) | 128.5251 |
| (C5,1N,H27) | 124.245 |
| (N1,C2,C3) | 105.2442 |
| (N1,C2,H28) | 122.3209 |
| (C3,C2,H28) | 132.4349 |
| (C2,C3,C4) | 110.735 |
| (C2,C3,H29) | 127.7322 |
| (C4,C3,H29) | 121.5329 |
| (C3,C4,C5) | 105.0196 |
| (N1,C5,C4) | 111.7713 |
| (N1,C5,C6) | 122.4229 |
| (C4,C5,C6) | 125.8058 |
| (C5,C6,N7) | 126.0913 |
| (C5,C6,N10) | 121.7341 |
| (N7,C6,N10) | 112.1746 |
| (C6,N7,C8) | 105.4895 |
| (N7,C8,C9) | 110.1349 |
| (C7,C8,C11) | 122.6324 |
| (C9,C8,C11) | 127.2326 |
| (C8,C9,N10) | 104.4228 |
| (C8,C9,C12) | 130.6621 |
| (N10,C9,C12) | 124.9151 |
| (C6,N10,C9) | 107.7782 |
| (C6,N10,H30) | 123.7181 |
| (C9, N10,H30) | 128.5037 |
| (C8,C11,C14) | 123.8562 |
| (C8,C11,H31) | 116.0247 |
| (C14,C11,H31) | 120.119 |
| (C9,C12,C13) | 126.2095 |
| (C9,C12,H32) | 114.4356 |
| (C13,C12,H32) | 119.3549 |
| (C12,C13,C16) | 127.1925 |
| (C12,C13,H33) | 118.6522 |
| (C16,C13,H33) | 114.1553 |
| (C11,C14,C15) | 127.3384 |
| (C11,1C4,H34) | 116.8435 |
| (C15,C14,H34) | 115.8181 |
| (C14,C15,C22) | 118.7656 |
| (C14,C15,C26) | 123.6189 |
| (C22,C15,C26) | 117.6155 |
| (C13,C16,C17) | 118.8417 |
| (C13,C16C,21) | 123.5118 |
| (C17,C16,C21) | 117.6465 |
| (C16,C17,C18) | 121.3697 |
| (C16,C17,H35) | 119.0078 |
| (C18,C17,H35) | 119.6225 |
| (C17,C18,C19) | 120.1534 |
| (C17,C18,H36) | 119.7082 |
| (C19,1C8,H36) | 120.1384 |
| (C18,C19,C20) | 119.3081 |
| (C18,C19,H37) | 120.4039 |
| (C20,C19,H37) | 120.2881 |
| (C19,C20,C21) | 120.5573 |
| (C19,C20,H38) | 119.928 |
| (C21,C20,H38) | 119.5148 |
| (C16,C21,C20) | 120.9651 |
| (C16,C21,H39) | 120.016 |
| (C20,C21,H39) | 119.0189 |
| (C15,C22,C23) | 121.3913 |
| (C15,C22,H40) | 118.8556 |
| (C23,C22,H40) | 119.7532 |
| (C22,C23,C24) | 120.1389 |
| (C22,C23,H41) | 119.7475 |
| (C24,C23,H41) | 120.1136 |
| (C23,C24,C25) | 119.3224 |
| (C23,C24,H42) | 120.4005 |
| (C25,C24,H42) | 120.2772 |
| (C24,C25,C26) | 120.5146 |
| (C24,C25,H43) | 119.9143 |
| (C26,C25,H43) | 119.571 |
| (C15,C26,C25) | 121.0173 |
| (C15,C26,H44) | 119.9046 |
| (C25,C26,H44) | 119.0781 |
| (C5,N1,C2,C3) | -0.0033 |
| (C5,N1,C2,H28) | -180.0005 |
| (H27,N1,C2,C3) | 179.9935 |
| (H27,N1,C2,H28) | -0.0038 |
| (C2,N1,C5,C4) | -0.0018 |
| (C2,N1,C5,C6) | 180.0004 |
| (H27,N1,C5,N4) | -179.9986 |
| (H27,N1,C5,C6) | 0.0035 |
| (N1,C2,C3,N4) | 0.0071 |
| (N1,2,C3,HC29) | 180.0012 |
| (H28,C2,C3,N4) | 180.0039 |
| (H28,C2,C3,C29) | -0.002 |
| (C2,C3,N4,C5) | -0.0081 |
| (H29,C3,N4,C5) | -180.0026 |
| (C3,N4,C5,N1) | 0.006 |
| (C3,N4,C5,C6) | 180.0038 |
| (N1,C5,C6,N7) | -0.0028 |
| (N1,C5,C6,N10) | 179.9969 |
| (N4,C5,C6,N7) | -180.0004 |
| (N4,C5,C6,N10) | -0.0006 |
| (C5,C6,N7,C8) | 179.9968 |
| (N10,C6,N7,C8) | -0.003 |
| (C5,C6,N10,C9) | -179.9987 |
| (C5,C6,C10,H30) | -0.008 |
| (N7,C6,N10,C9) | 0.0011 |
| (N7,C6,N10,H30) | 179.9918 |
| (C6,N7,C8,C9) | 0.0037 |
| (C6,N7,C8,C11) | -179.9964 |
| (N7,C8,C9,N10) | -0.003 |
| (N7,C8,C9,C12) | 179.9914 |
| (C11,C8,C9,N10) | 179.997 |
| (C11,C8,C9,C12) | -0.0086 |
| (N7,C8,C11,C14) | 0.0077 |
| (C7,C8,C11,H31) | -179.9956 |
| (C9,C8,C11,C14) | -179.9924 |
| (C9,C8,C11,H31) | 0.0044 |
| (C8,C9,N10,C6) | 0.0012 |
| (C8,C9,N10,H30) | -179.9889 |
| (C12,C9,N10,C6) | -179.9936 |
| (C12,C9,N10,H30) | 0.0164 |
| (C8,C9,C12,C13) | 180.0077 |
| (C8,C9,C12,H32) | 0.0104 |
| (N10,C9,C12,C13) | 0.001 |
| (N10,C9,C12,H32) | 180.0037 |
| (C8,C11,C14,C15) | -180.0008 |
| (C8,C11,C14,H34) | 0.0047 |
| (H31,C11,C14,C15) | 0.0026 |
| (H31,C11,C14,H34) | -179.9919 |
| (C9,C12,C13,C16) | 179.9999 |
| (C9,C12,C13,H33) | -0.0026 |
| (H32,C12,C13,C16) | -0.0029 |
| (H32,C12,C13,H33) | 179.9946 |
| (C12,C13,C16,C17) | 179.963 |
| (C12,C13,C16,C21) | -0.0376 |
| (H33,C13,C16,C17) | -0.0346 |
| (H33,C13,C16,C21) | 179.9648 |
| (C11,C14,C15,C22) | -179.9486 |
| (C11,C14,C15,C26) | 0.0521 |
| (H34,C14,C15,C22) | 0.0459 |
| (H34,C14,C15,C26) | -179.9533 |
| (C14,C15,C22,C23) | 179.9968 |
| (C14,C15,C22,H40) | -0.0022 |
| (C26,C15,C22,C23) | -0.0039 |
| (C26,C15,C22,H40) | 179.9971 |
| (C14,C15,C26,C25) | -179.9969 |
| (C14,C15,C26,H44) | 0.0082 |
| (C22,C15,C26,C25) | 0.0039 |
| (C22,C15C,26,H44) | -179.991 |
| (C13,C16,C17,C18) | -179.9979 |
| (C13,C16,C17,H35) | 0.0006 |
| (C21,C16,C17,C18) | 0.0027 |
| (C21,C16,C17,H35) | -179.9987 |
| (C13,C16,C21,C20) | 179.9976 |
| (C13,C16,C21,H39) | -0.0047 |
| (C17,C16,C21,C20) | -0.0031 |
| (C17,C16,C21,H39) | 179.9947 |
| (C16,C17,C18,C19) | -0.0011 |
| (C16,C17,C18,H36) | 179.9987 |
| (H35,C17,C18,C19) | -179.9996 |
| (H35C,17,C18,H36) | 0.0002 |
| (C17,C18,C19,C20) | -0.0002 |
| (C17,C18,C19,H37) | -179.999 |
| (H36,C18,C19,C20) | 180 |
| (H36,C18,C19,H37) | 0.0013 |
| (C18,C19,C20,C21) | -0.0002 |
| (C18,C19,C20,H38) | -179.9978 |
| (H37,C19,C20,C21) | 179.9986 |
| (H37,C19C,20H,38) | 0.001 |
| (C19,C20,C21,C16) | 0.0019 |
| (C19,C20,C21,H39) | -179.9959 |
| (H38,C20,C21,C16) | 179.9995 |
| (H38,C20,C21,H39) | 0.0017 |
| (C15,C22,C23,C24) | 0.0012 |
| (C15,C22,C23,H41) | -179.9994 |
| (H40,C22,C23,C24) | -179.9999 |
| (H40,C22,C23,H41) | -0.0004 |
| (C22,C23,C24,C25) | 0.0017 |
| (C22,C23,C24,H42) | 179.9999 |
| (H41,C23,C24,C25) | -179.9977 |
| (H41,C23,C24,H42) | 0.0005 |
| (C23,C24,C25,C26) | -0.0018 |
| (C23,C24,C25,H43) | 179.9964 |
| (H42,C24,C25,C26) | -180 |
| (H42,C24,C25,H43) | -0.0018 |
| (C24,C25,C26,C15) | -0.0011 |
| (C24,C25,C26,H44) | 179.9939 |
| (H43,C25,C26,C15) | -179.9992 |
| (H43,C25,C26,H44) | -0.0043 |

**Table S2 Natural bond orbital analysis of 4,5-bis [(E)-2-phenylethenyl]-1H,1’H-2,2’-biimidazole**

| **Donor** | **Type** | **ED/e** | **Acceptor** | **Type** | **ED/e** | **E(2)^a^** | **E(j-i)^b^** | **F(i,j)^c^** |
| --- | --- | --- | --- | --- | --- | --- | --- | --- |
| N 1 - C 2 | σ | 1.98393 | N 1 - C 5 | σ* | 0.04061 | 2.32 | 1.26 | 0.049 |
|  | σ |  | N 1 - H 27 | σ* | 0.01934 | 0.88 | 1.21 | 0.029 |
|  | σ |  | C 2 - C 3 | π* | 0.30853 | 0.77 | 1.4 | 0.029 |
|  | σ |  | C 3 - N 4 | σ* | 0.00982 | 0.93 | 1.29 | 0.031 |
|  | σ |  | C 3 - H 29 | σ* | 0.01559 | 3.61 | 1.27 | 0.061 |
|  | σ |  | N 4 - C 5 | π* | 0.44602 | 0.59 | 1.34 | 0.025 |
|  | σ |  | C 5 - C 6 | σ* | 0.03777 | 4.6 | 1.35 | 0.071 |
| N 1 - C 5 | σ | 1.9857 | N 1 - C 2 | σ* | 0.01335 | 1.9 | 1.27 | 0.044 |
|  | σ |  | N 1 - H 27 | σ* | 0.01934 | 1.07 | 1.24 | 0.032 |
|  | σ |  | C 2 - H 28 | σ* | 0.01198 | 2.82 | 1.28 | 0.054 |
|  | σ |  | C 3 - H 29 | σ* | 0.01559 | 0.58 | 1.3 | 0.025 |
|  | σ |  | N 4 - C 5 | π* | 0.44602 | 0.57 | 1.37 | 0.025 |
|  | σ |  | C 5 - C 6 | σ* | 0.03777 | 1.75 | 1.38 | 0.044 |
|  | σ |  | C 6 - N 10 | σ* | 0.03788 | 2.33 | 1.29 | 0.049 |
| N 1 - H 27 | σ | 1.99002 | N 1 - C 5 | σ* | 0.04061 | 0.51 | 1.15 | 0.022 |
|  | σ |  | C 2 - C 3 | π* | 0.30853 | 1.21 | 1.29 | 0.035 |
| N 1 - H 27 | σ |  | N 4 - C 5 | π* | 0.44602 | 2.4 | 1.23 | 0.049 |
| C 2 - C 3 | σ | 1.98459 | N 1 - C 2 | σ* | 0.01335 | 0.5 | 1.15 | 0.022 |
|  | σ |  | N 1 - H 27 | σ* | 0.01934 | 4.79 | 1.12 | 0.065 |
|  | σ |  | C 2 - H 28 | σ* | 0.01198 | 1.78 | 1.16 | 0.041 |
|  | σ |  | C 3 - H 29 | σ* | 0.01559 | 1.45 | 1.18 | 0.037 |
|  | σ |  | C 5 - C 6 | σ* | 0.03777 | 1.54 | 1.26 | 0.039 |
| C 2 - C 3 | σ | 1.83022 | N 4 - C 5 | π* | 0.44602 | 13.36 | 0.28 | 0.059 |
| C 2 - H 28 | σ | 1.98616 | N 1 - C 5 | σ* | 0.04061 | 3.08 | 1.02 | 0.05 |
|  | σ |  | C 2 - C 3 | π* | 0.30853 | 1.09 | 1.15 | 0.032 |
|  | σ |  | C 3 - N 4 | σ* | 0.00982 | 2.02 | 1.05 | 0.041 |
| C 3 - N 4 | σ | 1.97802 | N 1 - C 2 | σ* | 0.01335 | 1.16 | 1.2 | 0.033 |
|  | σ |  | N 1 - C 5 | σ* | 0.04061 | 1.11 | 1.22 | 0.033 |
|  | σ |  | C 2 - C 3 | π* | 0.30853 | 0.88 | 1.36 | 0.031 |
|  | σ |  | C 2 - H 28 | σ* | 0.01198 | 3.69 | 1.21 | 0.06 |
|  | σ |  | N 4 - C 5 | π* | 0.44602 | 1 | 1.3 | 0.032 |
|  | σ |  | C 5 - C 6 | σ* | 0.03777 | 7.39 | 1.31 | 0.088 |
| C 3 - H 29 | σ | 1.98125 | N 1 - C 2 | σ* | 0.01335 | 2.21 | 0.97 | 0.041 |
|  | σ |  | C 2 - C 3 | π* | 0.30853 | 0.59 | 1.13 | 0.023 |
|  | σ |  | N 4 - C 5 | σ* | 0.01577 | 3.14 | 1.07 | 0.052 |
| N 4 - C 5 | σ | 1.98125 | N 1 - C 5 | σ* | 0.04061 | 0.66 | 1.28 | 0.026 |
|  | σ |  | N 1 - H 27 | σ* | 0.01934 | 2.77 | 1.24 | 0.052 |
|  | σ |  | C 3 - N 4 | σ* | 0.00982 | 0.87 | 1.31 | 0.03 |
|  | σ |  | C 3 - H 29 | σ* | 0.01559 | 3.39 | 1.3 | 0.059 |
|  | σ |  | C 5 - C 6 | σ* | 0.03777 | 2.56 | 1.38 | 0.053 |
|  | σ |  | C 6 - N 7 | π* | 0.43088 | 2.15 | 1.37 | 0.049 |
| N 4 - C 5 | σ | 1.82038 | C 2 - C 3 | π* | 0.30853 | 22.26 | 0.33 | 0.079 |
|  | σ |  | C 6 - N 7 | π* | 0.43088 | 14.18 | 0.31 | 0.064 |
| C 5 - C 6 | σ | 1.97337 | N 1 - C 2 | σ* | 0.01335 | 2.13 | 1.16 | 0.044 |
|  | σ |  | N 1 - C 5 | σ* | 0.04061 | 0.9 | 1.17 | 0.029 |
|  | σ |  | C 3 - N 4 | σ* | 0.00982 | 2.22 | 1.2 | 0.046 |
|  | σ |  | N 4 - C 5 | π* | 0.44602 | 2.17 | 1.25 | 0.047 |
|  | σ |  | C 6 - N 7 | π* | 0.43088 | 2.22 | 1.26 | 0.047 |
|  | σ |  | C 6 - N 10 | σ* | 0.03788 | 0.89 | 1.17 | 0.029 |
|  | σ |  | N 7 - C 8 | σ* | 0.0192 | 2.33 | 1.19 | 0.047 |
|  | σ |  | C 9 - N 10 | σ* | 0.02543 | 2.26 | 1.15 | 0.046 |
| C 6 - N 7 | σ | 1.98018 | N 4 - C 5 | π* | 0.44602 | 2.07 | 1.37 | 0.048 |
|  | σ |  | C 5 - C 6 | σ* | 0.03777 | 2.45 | 1.38 | 0.052 |
|  | σ |  | C 6 - N 10 | σ* | 0.03788 | 0.68 | 1.29 | 0.027 |
|  | σ |  | N 7 - C 8 | σ* | 0.0192 | 1.07 | 1.31 | 0.033 |
|  | σ |  | C 8 - C 11 | σ* | 0.02491 | 4.87 | 1.37 | 0.073 |
|  | σ |  | N 10 - H 30 | σ* | 0.02017 | 2.72 | 1.24 | 0.052 |
|  | σ |  | N 4 - C 5 | π* | 0.44602 | 12.86 | 0.31 | 0.061 |
|  | σ |  | C 8 - C 9 | π* | 0.41438 | 21.12 | 0.34 | 0.08 |
| C 6 - N 10 | σ | 1.98392 | N 1 - C 5 | σ* | 0.04061 | 2.44 | 1.28 | 0.05 |
|  | σ |  | C 5 - C 6 | σ* | 0.03777 | 1.66 | 1.37 | 0.043 |
|  | σ |  | C 6 - N 7 | π* | 0.43088 | 0.62 | 1.37 | 0.026 |
|  | σ |  | C 8 - C 11 | σ* | 0.02491 | 0.92 | 1.37 | 0.032 |
|  | σ |  | C 9 - N 10 | σ* | 0.02543 | 2.15 | 1.26 | 0.047 |
|  | σ |  | C 9 - C 12 | σ* | 0.02224 | 4.32 | 1.37 | 0.069 |
|  | σ |  | N 10 - H 30 | σ* | 0.02017 | 1.15 | 1.23 | 0.034 |
| N 7 - C 8 | σ | 1.97446 | C 5 - C 6 | σ* | 0.03777 | 7.05 | 1.31 | 0.086 |
|  | σ |  | C 6 - N 7 | π* | 0.43088 | 1.18 | 1.31 | 0.035 |
|  | σ |  | C 6 - N 10 | σ* | 0.03788 | 0.95 | 1.22 | 0.031 |
|  | σ |  | C 8 - C 9 | π* | 0.41438 | 1.32 | 1.35 | 0.038 |
|  | σ |  | C 8 - C 11 | σ* | 0.02491 | 1.59 | 1.31 | 0.041 |
|  | σ |  | C 9 - N 10 | σ* | 0.02543 | 0.97 | 1.2 | 0.031 |
|  | σ |  | C 9 - C 12 | σ* | 0.02224 | 3.88 | 1.31 | 0.064 |
|  | σ |  | C 11 - H 31 | σ* | 0.01794 | 1.53 | 1.22 | 0.039 |
| C 8 - C 9 | σ | 1.97131 | C 5 - C 6 | σ* | 0.03777 | 1.2 | 1.24 | 0.035 |
|  | σ |  | N 7 - C 8 | σ* | 0.0192 | 0.65 | 1.17 | 0.025 |
|  | σ |  | C 8 - C 11 | σ* | 0.02491 | 3.51 | 1.23 | 0.059 |
|  | σ |  | C 9 - N 10 | σ* | 0.02543 | 0.7 | 1.13 | 0.025 |
|  | σ |  | C 9 - C 12 | σ* | 0.02224 | 4.46 | 1.24 | 0.066 |
|  | σ |  | N 10 - H 30 | σ* | 0.02017 | 4.69 | 1.1 | 0.064 |
|  | σ |  | C 11 - C 14 | π* | 0.15517 | 1.87 | 1.37 | 0.045 |
|  | σ |  | C 12 - C 13 | π* | 0.18955 | 2.08 | 1.35 | 0.048 |
| C 8 - C 9 | σ | 1.68064 | C 6 - N 7 | π* | 0.43088 | 13.8 | 0.26 | 0.056 |
|  | σ |  | C 11 - C 14 | π* | 0.15517 | 14.2 | 0.31 | 0.062 |
|  | σ |  | C 12 - C 13 | π* | 0.18955 | 17.3 | 0.3 | 0.066 |
| C 8 - C 11 | σ | 1.9741 | C 6 - N 7 | σ* | 0.43088 | 2.02 | 1.22 | 0.044 |
|  | σ |  | N 7 - C 8 | σ* | 0.0192 | 1.4 | 1.14 | 0.036 |
|  | σ |  | C 8 - C 9 | π* | 0.41438 | 3.4 | 1.25 | 0.058 |
|  | σ |  | C 9 - N 10 | σ* | 0.02543 | 1.46 | 1.11 | 0.036 |
|  | σ |  | C 11 - C 14 | π* | 0.15517 | 3.85 | 1.35 | 0.064 |
|  | σ |  | C 11 - H 31 | σ* | 0.01794 | 0.68 | 1.12 | 0.025 |
|  | σ |  | C 14 - C 15 | σ* | 0.02351 | 3.72 | 1.19 | 0.06 |
| C 9 - N 10 | σ | 1.98035 | C 5 - C 6 | σ* | 0.03777 | 4.17 | 1.35 | 0.067 |
|  | σ |  | C 6 - N 10 | σ* | 0.03788 | 2.47 | 1.26 | 0.05 |
|  | σ |  | N 7 - C 8 | σ* | 0.0192 | 0.72 | 1.28 | 0.027 |
|  | σ |  | C 8 - C 9 | π* | 0.41438 | 1.12 | 1.39 | 0.035 |
|  | σ |  | C 8 - C 11 | σ* | 0.02491 | 4.06 | 1.34 | 0.066 |
|  | σ |  | C 9 - C 12 | σ* | 0.02224 | 1.44 | 1.34 | 0.039 |
|  | σ |  | N 10 - H 30 | σ* | 0.02017 | 0.98 | 1.21 | 0.031 |
|  | σ |  | C 12 - H 32 | σ* | 0.01772 | 1.33 | 1.25 | 0.036 |
| C 9 - C 12 | σ | 0.97453 | C 6 - N 10 | σ* | 0.03788 | 1.93 | 1.15 | 0.042 |
|  | σ |  | N 7 - C 8 | σ* | 0.0192 | 1.36 | 1.17 | 0.036 |
|  | σ |  | C 8 - C 9 | π* | 0.41438 | 4.47 | 1.28 | 0.068 |
|  | σ |  | C 9 - N 10 | σ* | 0.02543 | 1.37 | 1.13 | 0.035 |
|  | σ |  | C 12 - C 13 | π* | 0.18955 | 3.9 | 1.35 | 0.065 |
|  | σ |  | C 12 - H 32 | σ* | 0.01772 | 0.74 | 1.14 | 0.026 |
|  | σ |  | C 13 - C 16 | σ* | 0.02357 | 3.44 | 1.21 | 0.058 |
| N 10 - H 30 | σ | 1.98884 | C 6 - N 7 | π* | 0.43088 | 2.45 | 1.24 | 0.049 |
|  | σ |  | C 6 - N 10 | σ* | 0.03788 | 0.53 | 1.15 | 0.022 |
|  | σ |  | C 8 - C 9 | π* | 0.41438 | 1.65 | 1.28 | 0.041 |
|  | σ |  | C 9 - N 10 | σ* | 0.02543 | 0.58 | 1.13 | 0.023 |
| C 11 - C 14 | σ | 1.97891 | C 8 - C 9 | π* | 0.41438 | 2.97 | 1.3 | 0.056 |
|  | σ |  | C 8 - C 11 | σ* | 0.02491 | 3.6 | 1.26 | 0.06 |
|  | σ |  | C 11 - H 31 | σ* | 0.01794 | 1.83 | 1.17 | 0.041 |
|  | σ |  | C 14 - C 15 | σ* | 0.02351 | 3.17 | 1.24 | 0.056 |
|  | σ |  | C 14 - H 34 | σ* | 0.01761 | 1.5 | 1.18 | 0.038 |
|  | σ |  | C 15 - C 22 | π* | 0.37937 | 2.17 | 1.3 | 0.047 |
| C 11 - C 14 | σ | 1.85165 | C 8 - C 9 | π* | 0.41438 | 16.19 | 0.29 | 0.066 |
|  | σ |  | C 15 - C 22 | π* | 0.37937 | 13.28 | 0.3 | 0.06 |
| C 11 - H 31 | σ | 1.97376 | N 7 - C 8 | σ* | 0.0192 | 5.48 | 0.99 | 0.066 |
|  | σ |  | C 11 - C 14 | π* | 0.15517 | 1.24 | 1.19 | 0.034 |
|  | σ |  | C 14 - H 34 | σ* | 0.01761 | 4.86 | 0.97 | 0.062 |
| C 12 - C 13 | σ | 1.97907 | C 8 - C 9 | π* | 0.41438 | 3.11 | 1.32 | 0.057 |
|  | σ |  | C 9 - C 12 | σ* | 0.02224 | 3.73 | 1.28 | 0.062 |
|  | σ |  | C 12 - H 32 | σ* | 0.01772 | 1.89 | 1.18 | 0.042 |
|  | σ |  | C 13 - C 16 | σ* | 0.02357 | 3.28 | 1.24 | 0.057 |
|  | σ |  | C 13 - H 33 | σ* | 0.01721 | 1.45 | 1.17 | 0.037 |
|  | σ |  | C 16 - C 17 | π* | 0.38494 | 2.11 | 1.31 | 0.047 |
| C 12 - C 13 | σ | 1.85446 | C 8 - C 9 | π* | 0.41438 | 15.36 | 0.3 | 0.066 |
|  | σ |  | C 16 - C 17 | π* | 0.38494 | 13.14 | 0.31 | 0.061 |
| C 12 - H 32 | σ | 1.96908 | C 9 - N 10 | σ* | 0.02543 | 7.06 | 0.96 | 0.073 |
|  | σ |  | C 12 - C 13 | σ* | 0.01348 | 1.28 | 1.18 | 0.035 |
|  | σ |  | C 13 - H 33 | σ* | 0.01721 | 5.34 | 0.95 | 0.064 |
| C 13 - C 16 | σ | 1.97333 | C 9 - C 12 | σ* | 0.02224 | 3.5 | 1.2 | 0.058 |
|  | σ |  | C 12 - C 13 | π* | 0.18955 | 3.5 | 1.32 | 0.061 |
|  | σ |  | C 13 - H 33 | σ* | 0.01721 | 0.59 | 1.09 | 0.023 |
|  | σ |  | C 16 - C 17 | π* | 0.38494 | 2.6 | 1.23 | 0.051 |
|  | σ |  | C 16 - C 21 | σ* | 0.02692 | 2.93 | 1.24 | 0.054 |
|  | σ |  | C 17 - C 18 | σ* | 0.01486 | 2.44 | 1.25 | 0.05 |
|  | σ |  | C 20 - C 21 | π* | 0.30606 | 2.15 | 1.26 | 0.047 |
| C 13 - H 33 | σ | 1.9756 | C 12 - C 13 | π* | 0.18955 | 1.01 | 1.17 | 0.031 |
|  | σ |  | C 12 - H 32 | σ* | 0.01772 | 5.55 | 0.96 | 0.065 |
|  | σ |  | C 16 - C 21 | σ* | 0.02692 | 4.82 | 1.09 | 0.065 |
| C 14 - C 15 | σ | 1.9736 | C 8 - C 11 | σ* | 0.02491 | 3.21 | 1.19 | 0.055 |
|  | σ |  | C 11 - C 14 | π* | 0.15517 | 3.33 | 1.32 | 0.06 |
|  | σ |  | C 14 - H 34 | σ* | 0.01761 | 0.58 | 1.11 | 0.023 |
|  | σ |  | C 15 - C 22 | π* | 0.37937 | 2.6 | 1.23 | 0.051 |
|  | σ |  | C 15 - C 26 | σ* | 0.02719 | 2.95 | 1.23 | 0.054 |
|  | σ |  | C 22 - C 23 | σ* | 0.01491 | 2.51 | 1.25 | 0.05 |
|  | σ |  | C 25 - C 26 | π* | 0.30698 | 2.19 | 1.25 | 0.047 |
| C 14 - H 34 | σ | 1.97422 | C 11 - C 14 | π* | 0.15517 | 1.21 | 1.17 | 0.034 |
|  | σ |  | C 11 - H 31 | σ* | 0.01794 | 5.95 | 0.95 | 0.067 |
|  | σ |  | C 15 - C 26 | σ* | 0.02719 | 4.88 | 1.08 | 0.065 |
| C 15 - C 22 | σ | 1.97171 | C 11 - C 14 | π* | 0.15517 | 2.34 | 1.35 | 0.05 |
|  | σ |  | C 14 - C 15 | σ* | 0.02351 | 2.65 | 1.2 | 0.05 |
|  | σ |  | C 15 - C 26 | σ* | 0.02719 | 3.54 | 1.26 | 0.06 |
|  | σ |  | C 22 - C 23 | σ* | 0.01491 | 2.77 | 1.28 | 0.053 |
|  | σ |  | C 22 - H 40 | σ* | 0.01443 | 1.04 | 1.13 | 0.031 |
|  | σ |  | C 23 - H 41 | σ* | 0.01398 | 2.53 | 1.14 | 0.048 |
|  | σ |  | C 26 - H 44 | σ* | 0.01489 | 2.77 | 1.14 | 0.05 |
| C 15 - C 22 | σ | 1.61923 | C 11 - C 14 | π* | 0.15517 | 15.96 | 0.29 | 0.065 |
|  | σ |  | C 23 - C 24 | π* | 0.33696 | 21.48 | 0.28 | 0.07 |
|  | σ |  | C 25 - C 26 | π* | 0.30698 | 19.05 | 0.28 | 0.067 |
| C 15 - C 26 | σ | 1.97103 | C 14 - C 15 | σ* | 0.02351 | 3.04 | 1.2 | 0.054 |
|  | σ |  | C 14 - H 34 | σ* | 0.01761 | 1.49 | 1.14 | 0.037 |
|  | σ |  | C 15 - C 22 | π* | 0.37937 | 3.58 | 1.26 | 0.06 |
|  | σ |  | C 22 - H 40 | σ* | 0.01443 | 2.58 | 1.13 | 0.048 |
|  | σ |  | C 25 - C 26 | π* | 0.30698 | 2.97 | 1.28 | 0.055 |
|  | σ |  | C 25 - H 43 | σ* | 0.01398 | 2.5 | 1.14 | 0.048 |
|  | σ |  | C 26 - H 44 | σ* | 0.01489 | 1.1 | 1.14 | 0.032 |
| C 16 - C 17 | σ | 1.9716 | C 12 - C 13 | π* | 0.18955 | 2.4 | 1.34 | 0.051 |
|  | σ |  | C 13 - C 16 | σ* | 0.02357 | 2.64 | 1.19 | 0.05 |
|  | σ |  | C 16 - C 21 | σ* | 0.02692 | 3.59 | 1.26 | 0.06 |
|  | σ |  | C 17 - C 18 | σ* | 0.01486 | 2.78 | 1.28 | 0.053 |
|  | σ |  | C 17 - H 35 | σ* | 0.01447 | 1.04 | 1.13 | 0.031 |
|  | σ |  | C 18 - H 36 | σ* | 0.01388 | 2.5 | 1.14 | 0.048 |
|  | σ |  | C 21 - H 39 | σ* | 0.01476 | 2.76 | 1.14 | 0.05 |
| C 16 - C 17 | σ | 1.62092 | C 12 - C 13 | π* | 0.18955 | 16.5 | 0.29 | 0.065 |
|  | σ |  | C 18 - C 19 | π* | 0.33571 | 21.31 | 0.28 | 0.07 |
|  | σ |  | C 20 - C 21 | π* | 0.30606 | 18.8 | 0.28 | 0.066 |
| C 16 - C 21 | σ | 1.97064 | C 13 - C 16 | σ* | 0.02357 | 3.06 | 1.19 | 0.054 |
|  | σ |  | C 13 - H 33 | σ* | 0.01721 | 1.55 | 1.12 | 0.037 |
|  | σ |  | C 16 - C 17 | π* | 0.38494 | 3.62 | 1.26 | 0.06 |
|  | σ |  | C 17 - H 35 | σ* | 0.01447 | 2.58 | 1.13 | 0.049 |
|  | σ |  | C 20 - C 21 | π* | 0.30606 | 2.96 | 1.28 | 0.055 |
|  | σ |  | C 20 - H 38 | σ* | 0.01387 | 2.47 | 1.14 | 0.048 |
|  | σ |  | C 21 - H 39 | σ* | 0.01476 | 1.09 | 1.14 | 0.032 |
| C 17 - C 18 | σ | 1.9783 | C 13 - C 16 | σ* | 0.02357 | 3.58 | 1.21 | 0.059 |
|  | σ |  | C 16 - C 17 | π* | 0.38494 | 3.62 | 1.27 | 0.056 |
|  | σ |  | C 17 - H 35 | σ* | 0.01447 | 1.22 | 1.14 | 0.033 |
|  | σ |  | C 18 - C 19 | π* | 0.33571 | 2.76 | 1.28 | 0.053 |
|  | σ |  | C 18 - H 36 | σ* | 0.01388 | 1.12 | 1.15 | 0.032 |
|  | σ | 1.98059 | C 19 - H 37 | σ* | 0.01383 | 2.72 | 1.15 | 0.05 |
| C 17 - H 35 | σ |  | C 16 - C 17 | π* | 0.38494 | 3.62 | 1.09 | 0.024 |
|  | σ |  | C 16 - C 21 | σ* | 0.02692 | 4.56 | 1.09 | 0.063 |
|  | σ |  | C 17 - C 18 | σ* | 0.01486 | 0.67 | 1.11 | 0.024 |
|  | σ |  | C 18 - C 19 | π* | 0.33571 | 4.06 | 1.1 | 0.06 |
| C 18 - C 19 | σ | 1.9783 | C 17 - C 18 | σ* | 0.01486 | 2.79 | 1.29 | 0.054 |
|  | σ |  | C 17 - H 35 | σ* | 0.01447 | 2.77 | 1.14 | 0.05 |
|  | σ |  | C 18 - H 36 | σ* | 0.01388 | 1.13 | 1.15 | 0.032 |
|  | σ |  | C 19 - C 20 | σ* | 0.01687 | 2.57 | 1.28 | 0.051 |
|  | σ |  | C 19 - H 37 | σ* | 0.01383 | 1.12 | 1.15 | 0.032 |
|  | σ |  | C 20 - H 38 | σ* | 0.01387 | 2.74 | 1.15 | 0.05 |
| C 18 - C 19 | π | 1.66 | C 16 - C 17 | π* | 0.38494 | 20.18 | 0.29 | 0.069 |
|  | σ |  | C 20 - C 21 | π* | 0.30606 | 20.28 | 0.29 | 0.069 |
| C 18 - H 36 | σ | 1.98063 | C 16 - C 17 | π* | 0.38494 | 4.41 | 1.09 | 0.062 |
|  | σ |  | C 17 - C 18 | σ* | 0.01486 | 0.65 | 1.11 | 0.024 |
|  | σ |  | C 18 - C 19 | π* | 0.33571 | 0.59 | 1.1 | 0.023 |
|  | σ |  | C 19 - C 20 | σ* | 0.01687 | 4 | 1.1 | 0.059 |
| C 19 - C 20 | σ | 1.97831 | C 18 - C 19 | π* | 0.33571 | 2.58 | 1.28 | 0.051 |
|  | σ |  | C 18 - H 36 | σ* | 0.01388 | 2.8 | 1.15 | 0.051 |
|  | σ |  | C 19 - H 37 | σ* | 0.01383 | 1.09 | 1.15 | 0.032 |
|  | σ |  | C 20 - C 21 | π* | 0.30606 | 2.76 | 1.29 | 0.053 |
|  | σ |  | C 20 - H 38 | σ* | 0.01387 | 1.09 | 1.15 | 0.032 |
|  | σ |  | C 21 - H 39 | σ* | 0.01476 | 2.72 | 1.14 | 0.05 |
| C 19 - H 37 | σ | 1.98127 | C 17 - C 18 | σ* | 0.01486 | 4.04 | 1.11 | 0.06 |
|  | σ |  | C 18 - C 19 | π* | 0.33571 | 0.61 | 1.1 | 0.023 |
|  | σ |  | C 19 - C 20 | σ* | 0.01687 | 0.58 | 1.1 | 0.023 |
|  | σ |  | C 20 - C 21 | π* | 0.30606 | 4.02 | 1.12 | 0.06 |
| C 20 - C 21 | σ | 1.97789 | C 13 - C 16 | σ* | 0.02357 | 3.92 | 1.21 | 0.061 |
|  | σ |  | C 16 - C 21 | σ* | 0.02692 | 3.25 | 1.27 | 0.057 |
|  | σ |  | C 19 - C 20 | σ* | 0.01687 | 2.73 | 1.28 | 0.053 |
|  | σ |  | C 19 - H 37 | σ* | 0.01383 | 2.63 | 1.16 | 0.049 |
|  | σ |  | C 20 - H 38 | σ* | 0.01387 | 1.12 | 1.15 | 0.032 |
|  | σ |  | C 21 - H 39 | σ* | 0.01476 | 1.3 | 1.15 | 0.035 |
| C 20 - C 21 | σ | 1.68768 | C 16 - C 17 | π* | 0.38494 | 19.54 | 0.29 | 0.068 |
|  | σ |  | C 18 - C 19 | π* | 0.33571 | 19.43 | 0.29 | 0.067 |
| C 20 - H 38 | σ | 1.98073 | C 16 - C 21 | σ* | 0.02692 | 4.5 | 1.09 | 0.063 |
|  | σ |  | C 18 - C 19 | π* | 0.33571 | 3.96 | 1.1 | 0.059 |
|  | σ |  | C 19 - C 20 | σ* | 0.01687 | 0.55 | 1.1 | 0.022 |
|  | σ |  | C 20 - C 21 | π* | 0.30606 | 0.67 | 1.11 | 0.024 |
| C 21 - H 39 | σ | 1.97974 | C 16 - C 17 | π* | 0.38494 | 4.44 | 1.09 | 0.062 |
|  | σ |  | C 16 - C 21 | σ* | 0.02692 | 0.73 | 1.09 | 0.025 |
|  | σ |  | C 19 - C 20 | σ* | 0.01687 | 4.19 | 1.1 | 0.061 |
|  | σ |  | C 20 - C 21 | π* | 0.30606 | 0.67 | 1.12 | 0.024 |
| C 22 - C 23 | σ | 1.97837 | C 14 - C 15 | σ* | 0.02351 | 3.51 | 1.21 | 0.058 |
|  | σ |  | C 15 - C 22 | π* | 0.37937 | 3.04 | 1.27 | 0.055 |
|  | σ |  | C 24 - H 42 | σ* | 0.0139 | 1.21 | 1.15 | 0.033 |
|  | σ |  | C 23 - C 24 | π* | 0.33696 | 2.78 | 1.28 | 0.053 |
|  | σ |  | C 23 - H 41 | σ* | 0.01398 | 1.12 | 1.15 | 0.032 |
|  | σ |  | C 24 - H 42 | σ* | 0.0139 | 2.74 | 1.15 | 0.05 |
| C 22 - H 40 | σ | 1.98059 | C 15 - C 22 | π* | 0.37937 | 0.66 | 1.09 | 0.024 |
|  | σ |  | C 15 - C 26 | σ* | 0.02719 | 4.57 | 1.09 | 0.063 |
|  | σ |  | C 22 - C 23 | σ* | 0.01491 | 0.66 | 1.11 | 0.024 |
|  | σ |  | C 23 - C 24 | π* | 0.33696 | 4.08 | 1.1 | 0.06 |
| C 23 - C 24 | σ | 1.9783 | C 23 - C 24 | π* | 0.33696 | 2.79 | 1.29 | 0.054 |
|  | σ |  | C 22 - H 40 | σ* | 0.01443 | 2.75 | 1.14 | 0.05 |
|  | σ |  | C 23 - H 41 | σ* | 0.01398 | 1.13 | 1.15 | 0.032 |
|  | σ |  | C 24 - C 25 | σ* | 0.01694 | 2.59 | 1.28 | 0.051 |
|  | σ |  | C 24 - H 42 | σ* | 0.0139 | 1.13 | 1.15 | 0.032 |
|  | σ |  | C 25 - H 43 | σ* | 0.01398 | 2.74 | 1.15 | 0.05 |
| C 23 - C 24 | σ | 1.66258 | C 15 - C 22 | π* | 0.37937 | 20.02 | 0.29 | 0.069 |
|  | σ |  | C 25 - C 26 | π* | 0.30698 | 20.18 | 0.29 | 0.069 |
| C 23 - H 41 | σ | 1.98076 | C 15 - C 22 | π* | 0.37937 | 4.37 | 1.09 | 0.062 |
|  | σ |  | C 23 - C 24 | π* | 0.33696 | 0.65 | 1.11 | 0.024 |
|  | σ |  | C 23 - C 24 | π* | 0.33696 | 0.58 | 1.1 | 0.023 |
|  | σ |  | C 24 - C 25 | σ* | 0.01694 | 4.01 | 1.1 | 0.059 |
| C 24 - C 25 | σ | 1.97826 | C 23 - C 24 | π* | 0.33696 | 2.6 | 1.28 | 0.052 |
|  | σ |  | C 23 - H 41 | σ* | 0.01398 | 2.8 | 1.15 | 0.051 |
|  | σ |  | C 24 - H 42 | σ* | 0.0139 | 1.1 | 1.15 | 0.032 |
|  | σ |  | C 25 - C 26 | π* | 0.30698 | 2.77 | 1.29 | 0.054 |
|  | σ |  | C 25 - H 43 | σ* | 0.01398 | 1.09 | 1.15 | 0.032 |
|  | σ |  | C 26 - H 44 | σ* | 0.01489 | 2.72 | 1.15 | 0.05 |
| C 24 - H 42 | σ | 1.98135 | C 22 - C 23 | σ* | 0.01491 | 4.03 | 1.11 | 0.06 |
|  | σ |  | C 23 - C 24 | π* | 0.33696 | 0.61 | 1.1 | 0.023 |
|  | σ |  | C 24 - C 25 | σ* | 0.01694 | 0.58 | 1.1 | 0.023 |
|  | σ |  | C 25 - C 26 | π* | 0.30698 | 4.01 | 1.12 | 0.06 |
| C 25 - C 26 | σ | 1.97803 | C 14 - C 15 | σ* | 0.02351 | 3.86 | 1.21 | 0.061 |
|  | σ |  | C 15 - C 26 | σ* | 0.02719 | 3.23 | 1.27 | 0.057 |
|  | σ |  | C 24 - H 42 | σ* | 0.0139 | 2.74 | 1.28 | 0.053 |
|  | σ |  | C 24 - H 42 | σ* | 0.0139 | 2.65 | 1.15 | 0.049 |
|  | σ |  | C 25 - H 43 | σ* | 0.01398 | 1.13 | 1.15 | 0.032 |
|  | σ |  | C 26 - H 44 | σ* | 0.01489 | 1.29 | 1.15 | 0.034 |
| C 25 - C 26 | π | 1.68765 | C 15 - C 22 | π* | 0.33696 | 19.42 | 0.29 | 0.068 |
|  | σ |  | C 23 - C 24 | π* | 0.33696 | 19.55 | 0.29 | 0.067 |
| C 25 - H 43 | σ | 1.98089 | C 23 - C 24 | π* | 0.33696 | 4.47 | 1.09 | 0.063 |
|  | σ |  | C 23 - C 24 | π* | 0.33696 | 3.96 | 1.1 | 0.059 |
|  | σ |  | C 24 - C 25 | σ* | 0.01694 | 0.55 | 1.1 | 0.022 |
|  | σ |  | C 25 - C 26 | π* | 0.30698 | 0.67 | 1.11 | 0.024 |
| C 26 - H 44 | σ | 1.99921 | C 15 - C 22 | π* | 0.37937 | 4.44 | 1.09 | 0.062 |
|  | σ |  | C 15 - C 26 | σ* | 0.02719 | 0.74 | 1.09 | 0.025 |
|  | σ |  | C 24 - C 25 | σ* | 0.01694 | 4.2 | 1.1 | 0.061 |
|  | σ |  | C 25 - C 26 | π* | 0.30698 | 0.66 | 1.12 | 0.024 |
| LP(1) | N1 | 1.56267 | C 2 - C 3 | π* | 0.30853 | 33.04 | 0.29 | 0.091 |
|  |  |  | N 4 - C 5 | π* | 0.44602 | 52.38 | 0.28 | 0.108 |
| LP(1) | N4 | 1.92465 | N 1 - C 5 | σ* | 0.04061 | 9.47 | 0.82 | 0.079 |
|  |  |  | N 1 - H 27 | σ* | 0.01934 | 0.57 | 0.77 | 0.019 |
|  |  |  | C 2 - C 3 | π* | 0.30853 | 5.8 | 0.96 | 0.067 |
|  |  |  | C 3 - H 29 | σ* | 0.01559 | 1.51 | 0.83 | 0.032 |
|  |  |  | C 5 - C 6 | σ* | 0.01559 | 0.76 | 0.91 | 0.024 |
| LP(1) | N7 | 1.9225 | C 5 - C 6 | σ* | 0.01559 | 0.85 | 0.91 | 0.025 |
|  |  |  | C 6 - N 10 | σ* | 0.03788 | 9.48 | 0.82 | 0.08 |
|  |  |  | C 8 - C 9 | π* | 0.41438 | 6.62 | 0.95 | 0.071 |
|  |  |  | C 8 - C 11 | σ* | 0.02491 | 0.84 | 0.9 | 0.025 |
|  |  |  | N 10 - H 30 | σ* | 0.02491 | 0.59 | 0.77 | 0.019 |
| LP(1) | N10 | 1.5795 | C 6 - N 7 | π* | 0.02491 | 54.07 | 0.28 | 0.11 |
|  |  |  | C 8 - C 9 | π* | 0.41438 | 34.64 | 0.3 | 0.091 |
|  |  |  | C 6 - N 7 | π* | 0.02491 | 54.07 | 0.28 | 0.11 |
|  |  |  | C 8 - C 9 | π* | 0.41438 | 34.64 | 0.3 | 0.091 |
